# Food-Derived Natural Compounds as Molecular Targets in Cancer Prevention

**DOI:** 10.3390/pharmaceutics18080978

**Published:** 2026-08-08

**Authors:** Megha Udayasankaran, Bhanu Shankar, Cheran Radhakrishnan, Sundar Raj Moorthy, Ramachandran Samivel, Ramachandran Vinayagam, Dhanavathy Gnanasampanthapandian, Kanagaraj Palaniyandi

**Affiliations:** 1 Cancer Science Laboratory, Department of Biotechnology, School of Bioengineering, SRM Institute of Science and Technology, Kattankulathur, Chennai 603203, Tamil Nadu, India; md5356@srmist.edu.in (M.U.); bhanuchowdary06@gmail.com (B.S.); cherankrish.20@gmail.com (C.R.); dhanavag@srmist.edu.in (D.G.); 2 Department of Biomedical Engineering, PPG Institute of Technology, Coimbatore 641035, Tamil Nadu, India; sundarrajbme.it@ppg.edu.in; 3 Department of Research, Saveetha College of Nursing (SCON), Saveetha Institute of Medical and Technical Sciences (SIMATS), Saveetha University, Thandalam, Chennai 602105, Tamil Nadu, India; ramvel82@gmail.com; 4 Department of Biotechnology, Yeungnam University, 280 Daehak-ro, Gyeongsan 38541, Republic of Korea

**Keywords:** bioactive natural compounds, cancer prevention, oncogenic signaling pathways, phytochemicals, nano delivery systems

## Abstract

Cancer prevention through dietary intervention utilizing bioactive natural compounds has garnered significant attention due to the therapeutic limitations of conventional cancer treatments. These plant-derived active compounds, including polyphenols, terpenoids, organosulfur compounds, bioactive peptides, and alkaloids, possess potent anticancer properties. This systemic review addresses a critical gap in the scientific literature by elucidating the precise multitargeted oncogenic regulatory mechanisms of these molecules. A comprehensive methodology was employed, involving a systematic literature search across major electronic databases (including PubMed, Web of Science, Embase, and SCOPUS) to identify relevant original peer-reviewed studies. The evidence gathered demonstrates that these active compounds deliver significant health benefits and protect cells by modulating crucial molecular targets involved in cell cycle regulation, apoptosis, oncogenic signaling, epigenetic control, angiogenesis, oxidative stress, and inflammation. Specifically, they operate via multi-targeted cascades, such as inhibiting the PI3K/Akt, NF-κB, and STAT3 pathways. To provide a clear structural overview, these active compounds are categorized comprehensively based on their botanical and structural origins, including spices, fruits, and rhizomes. However, despite their promising bioactivities, these compounds have not yet been fully translated into clinical therapy due to challenges such as low bioavailability, rapid metabolism, limited systematic exposure, and a lack of convincing evidence from large-scale clinical trials. Although most current evidence remains rooted in in vitro and experimental animal models, clinical validation through high-quality trials is still required. Ultimately, this review underscores the potential of these active compounds and highlights how advances in formulation and nano delivery strategies offer promising solutions for effective cancer prevention.

## 1. Introduction

Cancer remains a leading cause of morbidity and mortality globally, characterized by uncontrolled cellular proliferation, resistance to apoptosis, genomic instability, chronic inflammation, and metastatic progression [1]. Despite notable advancements in conventional cancer therapies, including chemotherapy, radiotherapy, and targeted pharmaceuticals, their clinical application is often restricted due to toxicity, drug resistance, high costs, and adverse side effects. These limitations have intensified the pursuit of safe, cost-effective, and preventive strategies, with growing interest in dietary and bioactive natural compounds [2].

Both epidemiological and experimental studies strongly indicate that diets rich in fruits, vegetables, whole grains, and bioactive components are closely associated with a reduced risk of various cancers. These compounds include a wide array of bioactive phytochemicals, such as polyphenols, terpenoids, organosulfur compounds, bioactive peptides, and alkaloids, which exhibit significant anticancer properties. Unlike synthetic drugs that target a single site, these natural compounds exert multi-targeted effects by modulating key molecular pathways involved in cancer initiation, promotion, and progression [3,4,5]. The bioactive compounds discussed in this review represent the most extensively studied classes of natural molecules, which have demonstrated cancer prevention effects by targeting molecular pathways essential for carcinogenesis. These compounds regulate oxidative stress, chronic inflammation, apoptosis, cell cycle progression, and epigenetic mechanisms. Experimental research provides substantial evidence of their cancer-prevention capabilities; however, human studies offer limited supportive evidence regarding the effectiveness of dietary intervention. This limitation arises from factors such as poor bioavailability, inter-individual metabolic variation, or differences in dietary intake, and the scarcity of well-designed clinical trials. A significant advantage of these active compounds is their ability to disrupt hallmark cancer processes, including dysregulated cell cycle control, evasion of apoptosis, sustained proliferative signaling, angiogenesis, metastasis, oxidative stress, and chronic inflammation. These compounds affect critical molecular targets, such as cyclins, cyclin-dependent kinases (CDKs), tumor suppressor proteins, apoptotic regulators, oncogenic signaling pathways, transcription factors, and epigenetic modifiers.

Recent evidence highlights the significance of bioactive compounds in epigenetic regulation, facilitating the long-term modulation of gene expression without altering the DNA sequence [6]. This review provides a comprehensive classification of bioactive compounds with anticancer potential, followed by an in-depth analysis of their molecular targets and mechanisms of action in cancer prevention. Additionally, it incorporates insights from omics-based approaches to validate their mechanistic roles. The review also addresses challenges related to bioavailability, metabolism, and clinical translation, emphasizing the need for advanced formulation and nanodelivery strategies to enhance therapeutic potential [2,5].

## 2. Methodology

The Preferred Reporting Items for Systematic Reviews and Meta-Analyses (PRISMA 2020) guidelines served as the foundation for this systematic review [7]. A comprehensive literature search was conducted across PubMed, Scopus, Web of Science, Embase, and Google Scholar to identify studies investigating the chemopreventive potential of food-derived natural compounds. The literature search was limited to English language articles published from January 2000 to March 2026. The search terms included “dietary phytochemicals,” “food-derived natural compounds,” “chemoprevention,” “cancer prevention,” “polyphenols,” “curcumin,” “resveratrol,” “quercetin,” “green tea polyphenols,” “lycopene,” “sulforaphane,” “bioactive peptides,” “alkaloids,” “cell cycle,” “apoptosis,” and “oxidative stress.” These terms were employed both individually and in combination.

Articles were selected for inclusion if they investigated food-derived natural compounds with anticancer or chemopreventive potential through in vitro experiments, animal models, translation research, or human clinical studies. Eligible study reports encompassed original research articles, systematic reviews, meta-analyses, and clinical trials that detailed molecular mechanisms, signaling pathways, therapeutic targets, bioavailability, delivery methods, and clinical outcomes. Conversely, exclusions were applied to conference abstracts, editorials, book chapters, duplicate publications, non-English foreign articles, and articles lacking sufficient experimental or mechanistic data.

After the removal of duplicate entries, the titles and abstracts of all retrieved papers were independently reviewed. Data extracted from each publication included the names of natural compounds under investigation, their source foods, the specific cancer types, experimental models, targeted signaling pathways and molecular targets, overall anti-tumor biological actions, and the level of evidence (with human involvement classified as phase II, III, IV or post-marketing). Additionally, the potential clinical phase for human trials and existing limitations were documented. The results were synthesized narratively and categorized into major classes of food-derived natural compounds, including polyphenols, carotenoids, organosulfur compounds, peptides, and alkaloids. Further categorization was based on primary molecular targets and biological actions, including regulation of the cell cycle and apoptosis, disruption of oncogenic signaling pathways such as PI3K/Akt, MAPK, NF-κB, STAT3, Wnt/β-catenin, modification of reactive oxygen species generation and inflammation, inhibition of angiogenesis, modulation of epigenetic processes, and the application of omics technologies. Priority was given to reports with a high level of evidence. Concurrently, limitations related to poor oral bioavailability, rapid metabolism, insufficient clinical evidence, and challenges in translational research were highlighted and evaluated to provide a comprehensive overview of current research and promising future directions in this field.

## 3. Classification of Food-Derived Natural Compounds with Anticancer Potential

### 3.1. Polyphenols

#### 3.1.1. Flavonoids (Quercetin and Catechin)

Quercetin, a polyphenolic flavonoid, is prevalent in fruits, vegetables, and medicinal plants. Numerous preclinical studies have highlighted its antioxidant, anti-inflammatory, immunomodulatory, and anticancer properties. Notably, quercetin selectively targets cancer cells while sparing normal cells in preclinical models, positioning it as a promising candidate for integrative cancer therapy. This review synthesizes findings from four major literature sources to provide a comprehensive overview of quercetin’s molecular mechanisms and therapeutic potential across various cancer types [8,9]. Quercetin demonstrates several physiological activities, including acting as an antioxidant and free radical scavenger, inhibiting inducible nitric oxide synthase (iNOS) and xanthine oxidase, reducing leukocyte immobilization, and modulating gene expression [10]. Numerous studies have shown quercetin’s efficacy in treating various diseases, such as coronary heart disease, diabetes, and cancer [11]. The polyphenolic structure of quercetin, characterized by five hydroxyl groups, confers strong redox-modulating activity. Despite its potent biological effects, quercetin encounters challenges such as poor water solubility, limited intestinal absorption, rapid metabolism, and low systemic bioavailability [12]. Preclinical and early clinical studies estimate its oral bioavailability to be ~5–10%. Isoquercitrin, an enzymatically modified form of quercetin, exhibits enhanced bioavailability and significant antiallergic effects, contributing to immune function [13]. This modified isoquercitrin is produced through a natural enzymatic process that attaches polysaccharides, converting quercetin into a water-soluble form (Alpha-Glycosyl Isoquercetin). Key benefits of quercetin include high absorption and increased bioavailability. Pharmacokinetic data indicate that isoquercetin’s absorption is up to 40 times higher (*C*_max_) than that of quercetin, reaching peak levels in the bloodstream within just 15 min [14].

A clinical study comprising 144 adenocarcinoma (AD) and 120 squamous cell carcinoma (SQ) patients revealed that a diet rich in quercetin significantly affects the expression of microRNAs (miRs), which regulate gene expression at the post-transcriptional level [15]. Notably, the Let-7 family and miR-146a, known for their tumor-suppressing properties, exhibit markedly higher expression in lung cancer patients with increased quercetin intake. In contrast, the oncogenic miR-17 is downregulated by 67% of its members [15]. A major therapeutic challenge is the low absorption of quercetin; thus, researchers are investigating methods to enhance its plasma concentrations, such as incorporating it into nanoparticles or modifying its chemical structure. The anticancer efficacy of nanoparticles was validated in a recent study by Lou et al., which showed that quercetin nanoparticles induced cell death in human neuroglioma cells in a dose- and time-dependent manner and significantly enhanced apoptosis in these cells [16].

Catechins, a class of flavan-3-ol polyphenols predominantly found in green tea (*Camellia sinensis*), have gained attention as potential agents for cancer prevention and treatment. Their polyphenolic structure allows for electron delocalization, which facilitates the neutralization of free radicals. Tea catechins, such as (−)-epigallocatechin-3-gallate (EGCG), have been shown to reduce reactive oxygen species (ROS) including superoxide radical, singlet oxygen, hydroxyl radical, peroxyl radical, nitric oxide, nitrogen dioxide, and peroxynitrite [17]. Among these, EGCG is the most biologically active and has been extensively researched. Catechins are prevalent in green tea and various plant-derived foods and beverages [18]. Following the consumption of green tea, human volunteers exhibited significantly higher salivary levels of EGCG, EGC, and (−)-epicatechin compared to their blood levels, whereas the same amount of green tea solids in capsule form resulted in undetectable salivary catechin levels [19]. The enzymatic conversion of EGCG to EGC was observed in the oral cavity. This enzyme activity, not previously documented, has been provisionally named catechin esterase or EGCG esterase. Human saliva is known to contain carboxylesterase-type esterases, which are inhibited by organophosphorus compounds [20]. However, the activity of salivary EGCG esterase was not inhibited by bis-4-nitrophenyl phosphate. Epidemiological studies have consistently linked high intake of catechin-rich diets with a reduced incidence of various cancers, including breast, prostate, colorectal, lung, and hematological malignancies [19]. EGCG and other tea catechins undergo extensive biotransformation [21]. Due to their catechol structure, EGCG and other catechins are readily methylated by catechol-*O*-methyltransferase as a detoxification process. In addition, catechins are glucuronidated by UDP-glucuronosyltransferases and sulfated by sulfotransferases. These enzymatic reactions primarily occur in the small intestine and liver, with multiple methylation and conjugation reactions possible on the same molecule [22]. Experimental evidence further supports their ability to disrupt key stages of carcinogenesis, including initiation, promotion, and progression [23]. Unlike conventional single-target anticancer drugs, catechins exhibit pleiotropic biological activity, enabling them to influence multiple signaling pathways involved in cancer cell survival, proliferation, and metastasis. Furthermore, catechins have shown the ability to enhance the efficacy of standard chemotherapeutic agents while mitigating their toxicity, highlighting their potential role as chemosensitizers [23,24].

#### 3.1.2. Stilbenes (Resveratrol)

Resveratrol (RSV) (C_14_H_12_O_3_; molecular weight 228.24 Da) belongs to the stilbene class of polyphenols, characterized by two phenolic rings connected via an ethylene bridge. It exists both in cis- and trans-isomeric forms, with the trans isomer being more stable and biologically active. Exposure to ultraviolet light can induce cis–trans isomerization, which has implications for formulation and storage. The phenolic hydroxyl groups confer antioxidant properties, and their planar structure facilitates interactions with various protein targets [25]. Vine plants produce RSV in significant quantities in response to biotic infections such as Botrytis cinerea, as well as abiotic stresses. RSV functions as a phytoalexin in vines, enhancing the natural defense mechanisms of grape plants. It is also present in other edible plants, including hops, peanuts, and various berries (e.g., blackberries, blackcurrants, blueberries, mulberries, and cranberries) [26]. RSV has been shown to suppress plasma insulin-like growth factor-1 (IGF-1) and insulin-like growth factor-binding protein 3 (IGFBP-3), which are proteins involved in the insulin signaling pathway associated with tumorigenesis [27]. Holcombe et al. [28] on dietary intervention in humans revealed that milligram doses of RSV from grapes inhibited wingless-related integration site (Wnt)-signaling, leading to an anti-proliferative effect. Moreover, in a phase I clinical trial, Nguyen et al. reported a significant reduction in Wnt target genes and stem cell markers in normal colonic mucosa [28,29].

In a study involving rats with 7,12-dimethylbenz[a]anthracene (DMBA)-induced mammary tumors, a well-established carcinogenesis model where DMBA is administered to induce mammary tumor formation [30]. Combination of RSV with the soy isoflavone genistein was found to be more effective than RSV alone in reducing tumor multiplicity and extending tumor latency. Additionally, oral administration of RSV was shown to decrease *N*-methyl-*N*-nitrosourea-induced tumorigenesis in rats. However, short-term prepubertal exposure to RSV resulted in endocrine disruption, as evidenced by a significant increase in irregular estrous cycles with an extended estrus phase, resulting in a higher incidence and multiplicity of mammary tumors in rats [29,31]. Unlike other anticancer drugs, RSV induces tumor cell death by modulating Fas and Fas ligand (FasL) levels. Fas (CD95) is a cell-surface death receptor and FasL is its binding partner; their interaction triggers the extrinsic apoptotic pathway. An in vitro study on RSV-induced apoptosis in multiple myeloma and T-cell leukemia cells emphasized the recruitment of Fas/CD95 signaling in lipid rafts, which is crucial in antimyeloma and antileukemia chemotherapy through the co-clustering of the Fas/CD95 death receptor and lipid rafts, while sparing normal lymphocytes [32]. Previous in vitro studies have reported this effect in leukemia cell lines, as well as in colon and breast carcinoma cells [31]. RSV possesses favorable physicochemical properties, including moderate lipophilicity and passive membrane permeability, which facilitate its intracellular accumulation and access to nuclear and mitochondrial targets [33].

The rhizome of *Curcuma longa*, commonly known as turmeric, and its powdered form contain curcumin, chemically identified as diferuloylmethane. This primary polyphenolic compound is extracted from the rhizome and is widely utilized as both a spice and a medicinal herb. The molecular structure of curcumin comprises two aromatic rings with ortho-methoxy phenolic groups connected by a seven-carbon α, β-unsaturated β-diketone bridge, which significantly enhances its electrophile scavenging and antioxidant capabilities [34]. This structural configuration allows curcumin to engage with cellular membranes, lipophilic compartments, and signaling proteins, which is essential for its chemopreventive properties [35]. Epidemiological studies suggest that populations consuming turmeric-rich diets exhibit lower incidences of gastrointestinal, breast, and prostate cancers, highlighting curcumin’s preventive potential [36]. Curcumin exhibits potent antioxidant properties by directly neutralizing ROS and reactive nitrogen species (RNS), thereby preventing oxidative DNA damage, lipid peroxidation, and protein oxidation, which are initial steps in carcinogenesis [37]. It also reduces tumor-promoting inflammation by inhibiting NF-κB and activator protein-1 (AP-1), thus suppressing downstream effectors such as cyclooxygenase-2 (COX-2), inducible nitric oxide synthase (iNOS), TNF-α, IL-1β, and IL-6 [38,39]. These anti-inflammatory effects are crucial in reducing the microenvironmental factors that facilitate tumor initiation and progression [40]. On a molecular signaling level, curcumin targets multiple oncogenic pathways simultaneously. In addition, curcumin acts synergistically with other bioactive compounds such as RSV, quercetin, and standard chemotherapeutics, enhancing apoptosis and overcoming drug resistance [38]. In vitro studies have demonstrated that curcumin sensitizes human and rat glioma cells to radiation therapy in T98G, U87MG, and T67 cells, and inhibit AP-1 and NF-κB signaling pathways. Curcumin at a concentration of 20 μM inhibited TPA-stimulated PKC activity in human astroglioma cells and downregulated pro-angiogenic AP-1 and MMP9. In human HCT-116 colon cancer cells, curcumin (10–25 μM) inhibited PKC activation by preventing the release of Ca^2+^ from the endoplasmic reticulum [39,41]. Another in vitro study revealed that curcumin suppressed JNK activation induced by carcinogens [42]. Curcumin inhibited hydrogen peroxide-stimulated proliferation of LnCap prostate cancer cells by suppressing the AP-1 transcription factor. Prusty and Das reported that curcumin downregulated AP-1 in cervical cancer cells [43]. Consequently, curcumin’s inhibition of PKC activity could inhibit neovascularization in tumors by disrupting pro-angiogenic signaling through the ERK-AP-1-MMP-9 pathway. In the DMBA-induced hamster buccal pouch model of carcinogenesis, a 1% dietary turmeric intake over 12 weeks reduced the DMBA-induced tumor burden by downregulating the *Ras* oncogene product p21 [39]. When Hepa1–6 cells were transfected with c-Met-CAT promoter constructs and subsequently stimulated with HGF, a rapid increase in AP-1 DNA binding activity was observed. However, the incubation of these cells with curcumin resulted in abrogation of c-Met promoter activity. Curcumin’s mechanisms, which encompass antioxidants, anti-inflammatory, apoptotic, autophagic, anti-proliferative, anti-angiogenic, and anti-metastatic activities, in conjunction with advanced delivery strategies, render it as a promising candidate for cancer chemoprevention and adjuvant therapy [44]. Despite the notable anti-cancer properties demonstrated by curcumin in numerous in vitro and animal studies, clinical evidence remains limited. Although early-stage clinical trials have confirmed its safety and bioactivity, randomized controlled trials that substantiate its effectiveness are still absent. Therefore, additional clinical studies are necessary to validate curcumin’s anti-cancer properties.

### 3.2. Carotenoids

#### 3.2.1. Carotenoids—Lycopene

Lycopene, a carotenoid that does not convert to vitamin A, is prevalent in red-hued fruits and vegetables, including tomatoes, watermelon, pink grapefruit, guava, and papaya, as well as in fresh and processed tomato products (e.g., sauce and paste). This compound is an acyclic carotene characterized by 11 conjugated double bonds, which confer strong antioxidant properties by neutralizing singlet oxygen and free radicals. Its lipophilic nature allows integration into cellular membranes, thereby protecting lipid bilayers, DNA, and proteins from oxidative damage, which is critical for preventing mutation accumulation and tumor development [45]. Epidemiological research consistently associates higher lycopene intake with a decreased risk of prostate, breast, lung, and gastrointestinal cancers, underscoring its importance in cancer prevention [46]. Lycopene imparts anti-proliferative effects by modulating various oncogenic signaling pathways, notably suppressing the PI3K/Akt/mTOR axis, which reduces cell proliferation and enhances apoptosis sensitivity [47]. Both preclinical and clinical studies support lycopene’s efficacy. In vitro studies demonstrate dose-dependent inhibition of proliferation and induction of apoptosis in prostate, breast, and colon cancer cell lines [48]. In vivo studies in rodents show that dietary lycopene reduces tumor incidence, size, and metastasis, especially in chemically induced carcinogenesis models [49]. Although limited, clinical trials suggest that lycopene supplementation lowers PSA levels in prostate cancer patients and reduces oxidative stress markers [50]. Lycopene acts synergistically with other dietary antioxidants, such as vitamin E, selenium, and polyphenols, enhancing its cancer-preventive effects. However, lycopene’s bioavailability is affected by dietary fat intake, dietary processing, and conversion to the cis isomers, which are more efficiently absorbed and biologically active [51]. Lycopene’s diverse mechanisms, including antioxidant, anti-inflammatory, apoptotic, anti-proliferative, anti-angiogenic, and anti-metastatic activities, combined with its favorable dietary availability and synergistic potential with other bioactive peptides, render it a promising agent for cancer prevention [52,53].

#### 3.2.2. Carotenoids—β-Carotene

Carrots, sweet potatoes, pumpkins, spinach, and kale exemplify whole vegetables and fruits rich in β-carotene, a carotenoid with provitamin A properties. This compound is prevalent in orange and dark green vegetables such as carrots, sweet potatoes, spinach, kale, and pumpkins. From a chemical perspective, β-carotene is a tetraterpene characterized by a sequence of conjugated double bonds and β-ionone rings at each terminus, facilitating its role as a potent antioxidant and singlet oxygen quencher [54]. Its lipophilic nature permits integration into cell membranes and lipoproteins, thereby protecting lipid bilayers and intracellular macromolecules from oxidative damage, a critical factor in tumor development [28]. Epidemiological research suggests that diets rich in fruits and vegetables containing β-carotene correlate with a decreased risk of lung, gastric, and esophageal cancers, highlighting their preventive role in human health [55]. β-carotene offers antioxidant and anti-proliferative advantages by neutralizing ROS, thus reducing DNA strand breaks, lipid peroxidation, and protein oxidation [55,56]. It can modulate gene expression through retinoid signaling; enzymatic cleavage by β-carotene 15,15′-monooxygenase yields retinal, which is subsequently converted into retinoic acid. Retinoic acid engages with nuclear receptors RAR and RXR, controlling genes associated with cell differentiation, proliferation, and apoptosis, thereby maintaining normal tissue homeostasis [57]. On a molecular signaling level, preclinical studies demonstrate that β-carotene inhibits oncogenic pathways such as PI3K/Akt and MAPK, reducing proliferation and promoting apoptosis in precancerous and cancerous cells. This underscores the significance of obtaining β-carotene from whole dietary sources rather than isolated high-dose supplements. In addition, β-carotene exhibits synergistic interactions with other dietary antioxidants such as vitamin E, selenium, and polyphenols, enhancing its chemopreventive potential by strengthening antioxidant defenses, modulating signaling pathways, and promoting apoptosis in preclinical models [58]. In summary, the diverse mechanisms of β-carotene, including its antioxidant, anti-inflammatory, apoptotic, anti-proliferative, immunomodulatory, and detoxifying effects, along with its dietary availability and synergy with other phytochemicals, reinforce its role as a chemopreventive agent, especially when sourced from natural dietary sources [59,60].

### 3.3. Organosulfur Compounds

#### 3.3.1. Sulforaphane

Cruciferous vegetables are abundant in naturally occurring compounds with chemoprotective and anti-cancer properties, notably isothiocyanates (ITCs). These compounds are produced through the enzymatic hydrolysis of glucosinolates, which are sulfur-containing secondary metabolites characteristic of the Cruciferae family [61]. Among these, sulforaphane is one of the most extensively studied natural compounds with anti-cancer properties. Glucosinolates typically consist of a β-D-thioglucose group, a sulfonated oxime group, and a variable side chain. The glucosinolate precursor to sulforaphane, glucoraphanin, is prevalent in broccoli, cauliflower, and cabbage [4]. When plant tissues are disrupted by chewing or processing, the enzyme myrosinase hydrolyzes glucosinolates into bioactive aglycones, which are responsible for the anticancer effects associated with the consumption of cruciferous vegetables. Sulforaphane exhibits multistage chemopreventive activity during both the initiation and progression phases of cancer. In the initial stage, sulforaphane functions as a blocking agent by inhibiting phase I enzymes such as cytochrome P450, which convert procarcinogens into reactive carcinogenic intermediates, while simultaneously inducing phase II detoxification enzymes. These effects are primarily mediated through the activation of the Nrf2-ARE signaling pathway, which regulates cellular antioxidant and detoxification processes [62]. Preclinical studies, including in vitro experiments, have shown that sulforaphane induces cell-cycle arrest, promotes apoptosis, and inhibits cell proliferation in cancer cells. It exerts epigenetic regulatory effects by modulating histone deacetylases and DNA methylation. Additionally, it influences key signaling pathways involved in tumor cell development, such as NF-κB, MAPK, and PI3K/Akt. Reinforcing dietary strategies with sulforaphane is important for cancer prevention [63].

#### 3.3.2. Allicin

Allicin, or diallylthiosulfinate, is a protective compound derived from garlic that demonstrates a wide range of biological functions. The enzyme allicinase catalyzes the synthesis of allicin, which engages in a redox reaction with the thiol group in glutathione. Allicin is active in microbial, plant, and mammalian cell lines, where it promotes cancer cell death and inhibits proliferation [64]. Due to its hydrophobic nature, allicin effectively penetrates cellular membranes and acts as a reactive sulfur species within cells. It acts as an antimicrobial agent against various microorganisms, including *Staphylococcus aureus*, *Helicobacter pylori*, and *Candida albicans*. Allicin inhibits several enzymes by interacting with cysteine residues, which can reduce triglycerides and low-density cholesterol in humans [65]. It suppresses abnormal cell growth by blocking key oncogenic signaling pathways such as PI3K/Akt, MAPK, and NF, through the downregulation of cyclin D1 and CDKs [66]. In vitro studies have shown that allicin induces cell-cycle arrest at the G0/G1, or G2/M phases, thereby preventing uncontrolled cell division. Understanding its growth-regulatory effects is crucial for cancer prevention [67]. In vitro studies have also shown that allicin plays a significant role in reinstating sensitivity to programmed cell death, a feature often lost in early tumorigenesis, by activating the intrinsic mitochondrial apoptotic pathway. In addition, allicin targets altered cellular energetics, a hallmark of increased glycolysis and metabolic reprogramming, by inhibiting glycolytic enzymes and modulating mitochondrial function. It exhibits potent antioxidant and anti-inflammatory effects, which are crucial for cancer prevention, involving enzymes such as glutathione peroxidase and superoxide dismutase [68].

### 3.4. Bioactive Peptides and Alkaloids

**Figure 1:**
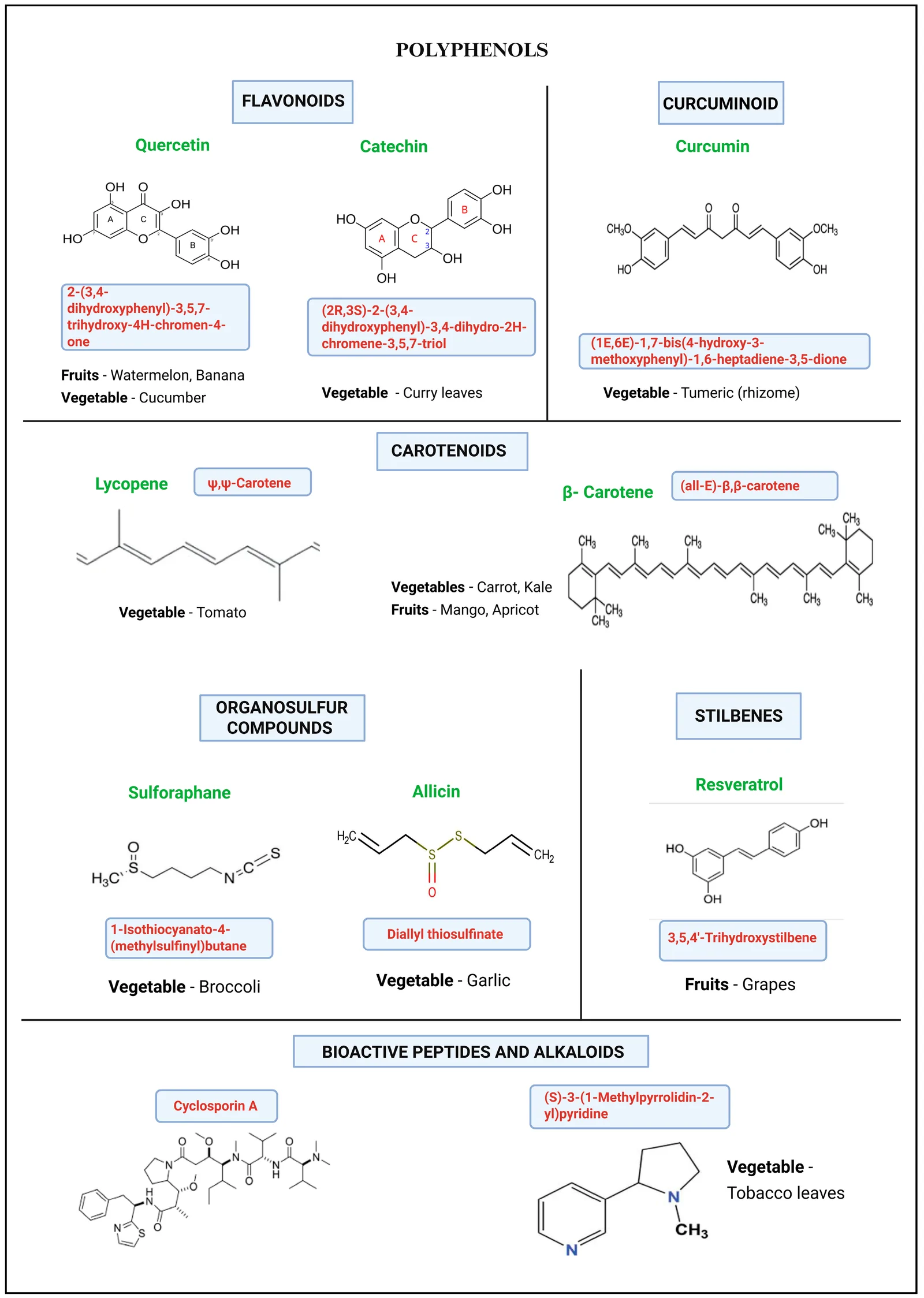
Chemical structure of natural compounds.

Bioactive peptides, which are short chains of amino acids, are derived from parent proteins found in plants, animals, marine organisms, and venoms, typically through enzymatic hydrolysis or fermentation. In recent years, these peptides have garnered significant attention due to their antioxidants, anti-inflammatory, and anticancer activities. Research has demonstrated that several bioactive peptides can inhibit cancer cell proliferation and induce apoptosis, thereby exhibiting antioxidants, anti-inflammatory, immunomodulatory, and anticancer activities [69]. Despite the promising effects of bioactive peptides on chronic diseases, there remains a paucity of clinical evidence regarding their role in cancer prevention. Consequently, further well-designed animal studies and randomized clinical trials are necessary. Conversely, alkaloids represent a broad class of naturally occurring organic compounds containing nitrogen atoms, predominantly found in plants. Many well-known chemotherapeutic drugs, such as vincristine and paclitaxel, have been developed from or inspired by alkaloids. Compounds such as vincristine, vinblastine, camptothecin, and berberine, distinct from alkaloids, target critical molecular pathways, including PI3K/Akt, MAPK, NF-κB, and caspase-dependent apoptotic cascades, which are instrumental in inducing cancer cell death [70]. The mechanism of action involves targeting molecular pathways, inducing apoptosis, causing cell cycle arrest, and inhibiting topoisomerase and microtubule assembly (Figure 1 and Table 1).

## 4. Regulation of Cell Cycle Checkpoints

### 4.1. Cyclins (Cyclin D, Cyclin E)

**Figure 2:**
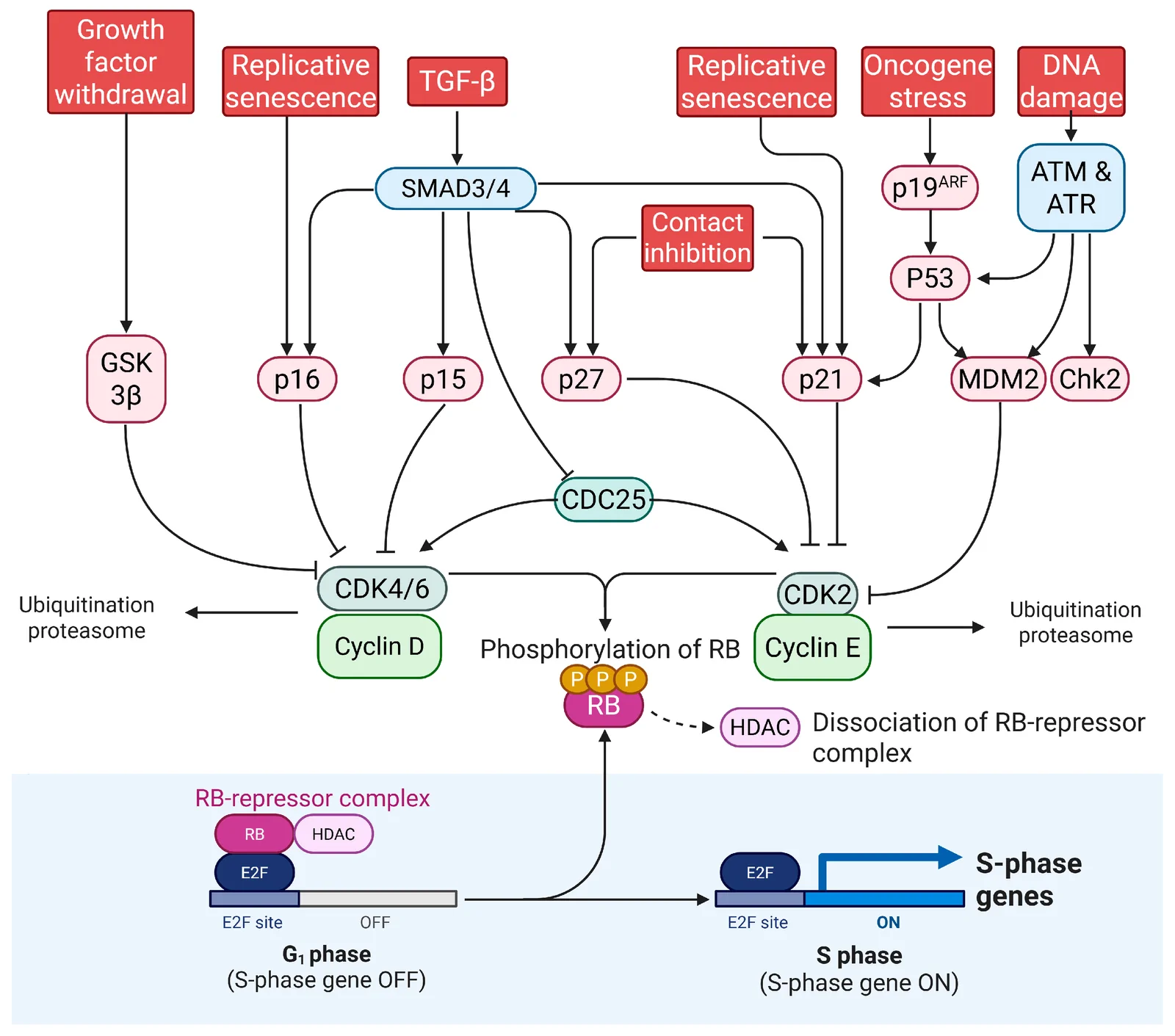
Cell cycle regulation.

Cyclins are proteins that regulate the cell cycle by activating cyclin-dependent kinases (CDKs), thereby facilitating the progression of the cell cycle through its various phases. Cyclin D and Cyclin E are particularly crucial for the regulation of the G1/S transition, a checkpoint often dysregulated in cancer (Figure 2). In breast cancer, Cyclin D1 is frequently overexpressed, which correlates with hormone-dependent tumor growth and poor prognosis. Similarly, elevated levels of Cyclin E are associated with genomic instability and more aggressive tumor characteristics. In colorectal cancer, aberrant Wnt/β-catenin signaling results in the transcriptional activation of Cyclin D1, whereas pancreatic cancer often shows Cyclin D1 amplification due to *KRAS* mutations [71].

Experimental studies have shown that polyphenols, including quercetin and catechins (EGCG), limit the downregulation of Cyclin D1 and Cyclin E at both transcriptional and protein levels, thereby disrupting the formation of cyclin–CDK complex. Furthermore, research has demonstrated that curcumin is particularly well-documented for its ability to suppress Cyclin D1 and Cyclin E expression across various cancer types. This suppression occurs through the inhibition of transcription factors such as NF-κB, AP-1, and STAT3, which are essential for cyclin gene expression in breast, colorectal, and pancreatic cancer cells. Such inhibition results in G0/G1 arrest, effectively halting uncontrolled cell growth. In vitro studies using breast cancer cell lines, such as MCF-7 and MDA-MB-231, have shown that curcumin significantly reduces Cyclin D1 levels, thereby limiting proliferation driven by estrogen and growth factors. Stilbenes, such as RSV, decrease Cyclin D1 expression by inhibiting upstream oncogenic pathways, including PI3K/Akt and MAPK/ERK (in vitro). The reduction in cyclin expression maintains retinoblastoma (Rb) in a hypophosphorylated, growth-inhibiting state, preventing E2F-mediated transcription of S-phase genes. This leads to G1 cell cycle arrest, decreased DNA synthesis, and inhibition of cancer cell proliferation in breast, oral, and colorectal cancer models.

Mechanistic studies demonstrate that sulforaphane enhances these outcomes by consistently activating the CDK inhibitors p21^cip^ p27^kip1^ and facilitating the proteasomal degradation of Cyclin D1, resulting in sustained G1 arrest in colorectal and pancreatic cancer models [72]. In addition, allicin contributes by modulating redox-sensitive signaling pathways and inhibiting cyclin induction mediated by MAPK and PI3K/Akt, thereby suppressing the formation of the Cyclin D–CDK4/6 complex [73,74].

Lycopene contributes to the regulation of cyclins by inhibiting the insulin-like growth factor-1 (IGF-1) signaling pathway, which is often activated in breast and colorectal cancers [75]. This inhibition results in decreased Cyclin D1 expression and reduced CDK activation. Similarly, β-carotene has been consistently observed to indirectly reduce cyclin expression by modulating redox-sensitive signaling pathways. Recent studies indicate that bioactive peptides and plant-derived alkaloids interfere with Cyclin D/E stability and CDK activity, thereby inducing cell cycle arrest in these tumor types. Collectively, these natural products target cyclin dysregulation at its molecular origin, preventing inappropriate cell cycle entry and consistently restricting tumor growth [76,77].

### 4.2. Cyclin-Dependent Kinases

CDKs act as the catalytic engines of the cell cycle, creating active complexes with cyclins to phosphorylate downstream targets such as the Rb protein. In malignancies, including breast, colorectal, and pancreatic cancers, CDKs (especially CDK2, CDK4, and CDK6) are often hyperactivated, resulting in the disruption of checkpoint control and unrestrained cellular proliferation. The hyperphosphorylation of Rb releases E2F transcription factors, which subsequently stimulate the expression of genes necessary for DNA synthesis and the transition into the S-phase [78,79].

Polyphenols, including quercetin and catechins, inhibit CDK2, CDK4, and CDK6 by directly blocking kinase activity and indirectly suppressing upstream signaling pathways such as PI3K/Akt and MAPK. Curcumin inhibits CDK activity through multiple mechanisms. It suppresses CDK expressions at the transcriptional level and consistently inhibits upstream signaling pathways, including PI3K/Akt and MAPK, which are crucial for CDK activation in cancer cells. In colorectal cancer models, curcumin treatment reduces CDK2 and CDK4 activity, resulting in reduced Rb phosphorylation and inhibition of E2F-mediated transcription. In pancreatic cancer, curcumin disrupts KRAS-driven CDK activation, thereby consistently slowing tumor cell growth [80]. Stilbenes, such as RSV, further decrease CDK activity by consistently disrupting cyclin–CDK complex formation. Reduced CDK activity prevents the phosphorylation of the Rb protein, maintaining Rb in its growth-inhibitory state. This blocks E2F-mediated transcription of S-phase genes, leading to G1 cell cycle arrest and reduced proliferation in breast and colorectal cancer models.

Sulforaphane operates synergistically with curcumin by consistently inhibiting CDK4/6 activity and promoting the proteasomal degradation of CDK–cyclin complexes. In addition, it epigenetically induces CDK inhibitor genes through histone acetylation and DNA demethylation, resulting in sustained checkpoint enhancement in breast and colorectal cancer models. Allicin further suppresses CDK signaling by consistently interfering with thiol-dependent kinase activity and inhibiting PI3K/Akt-driven CDK phosphorylation, especially in pancreatic cancer cells with high oxidative stress [81]. Recent studies suggest that bioactive peptides can influence CDK activity by disrupting kinase–substrate interactions, while plant-derived alkaloids directly target the ATP-binding sites of CDKs, imitating pharmacological CDK inhibitors [63,82].

β-Carotene contributes to the regulation of CDKs by modulating the intracellular redox balance. In cancer cells, oxidative stress is known to enhance CDK activation; however, the antioxidant properties of β-carotene mitigate this effect, especially in colorectal cancer. Similarly, lycopene disrupts CDK signaling by interfering with CDK activation via growth factors. Collectively, these bioactive natural compounds effectively inhibit CDK activity, thereby maintaining checkpoint integrity and preventing the progression of the malignant cell cycle [81,83].

### 4.3. CDK Inhibitors (p21, p27)

CDK inhibitors, specifically p21^cip^ p27^kip1^, serve as essential negative regulators of CDK activity by maintaining cell cycle checkpoints and inhibiting uncontrolled cell proliferation. In oncological contexts, these inhibitors frequently experience downregulation or inactivation, facilitating persistent CDK signaling. In breast cancer, reduced expression of p27 is associated with unfavorable prognoses, whereas colorectal and pancreatic cancers commonly exhibit disrupted regulation of p21 [84,85].

Polyphenols, including quercetin and EGCG, significantly enhance the expression of p21 and p27 through mechanisms that are both p53-dependent and independent. Curcumin has been shown to strongly induce the expression of p21 and p27 in various cancer models. Specifically, in breast cancer, curcumin increases levels of p21 through both p53-dependent and independent pathways, leading to an extended G1 phase arrest. In colorectal cancer cells, curcumin-induced p21 expression disrupts the activity of CDK2 and CDK4, thereby strengthening checkpoint control. In pancreatic cancer, curcumin upregulates p21 and p27 expression by inhibiting oncogenic signaling pathways, such as Akt and STAT3 [86].

Stilbenes, especially RSV, markedly enhance the levels of p21 and p27 during both transcriptional and post-translational stages [87]. The increased expression of these CDK inhibitors results in the suppression of CDK2, CDK4, and CDK6 activities, thereby maintaining the retinoblastoma (Rb) protein in a hypophosphorylated state that inhibits cellular growth. This state consistently prevents E2F-mediated transcription of S-phase genes, resulting in G1 cell cycle arrest. These effects have been consistently observed in models of breast, colorectal, and oral cancer.

Lycopene plays a role in stabilizing p27 by inhibiting its degradation via the proteasome, especially in breast and colorectal cancer cells [88]. The extended presence of p27 enhances the inhibition of CDKs, thereby preventing the re-initiation of the cell cycle. In addition, β-carotene contributes to the expression of CDK inhibitors by modulating oxidative stress and transcriptional regulation. Collectively, these natural compounds assist in restoring the balance between CDKs and their inhibitors, thereby reinforcing checkpoint control and inhibiting tumor cell proliferation [89].

Sulforaphane has been widely reported to induce the expression of p21 and p27 across various cancer models through mechanisms that are both p53-dependent and independent. In breast cancer cells, sulforaphane activates the Nrf2 pathway, thereby enhancing p21transcription and resulting in sustained G1 phase arrest, even in the absence of p53. In colorectal cancer, the sulforaphane-induced p21 expression inhibits CDK2 and CDK4 activities, resulting in Rb hypo-phosphorylation and the suppression of E2F-driven transcription. In pancreatic cancer, although KRAS-driven signaling reduces CDK inhibitor expression, sulforaphane counteracts this by restoring p21 and p27 levels through the inhibition of PI3K/Akt and STAT3 signaling pathways. This restoration reinforces checkpoint control, and restricts rapid tumor cell proliferation [63,90].

Allicin contributes to the regulation of CDK inhibitors through the modification of proteins containing thiol groups and the alteration of redox-sensitive pathways. By consistently reducing oxidative stress within cells and inhibiting Akt phosphorylation, allicin stabilizes p27, thereby preventing its cytoplasmic sequestration and proteasomal degradation, particularly in breast and pancreatic cancer cells. This stabilization facilitates the accumulation of p27 in the nucleus, thereby enhancing its inhibitory effect on CDKs. Furthermore, allicin-induced mitochondrial stress activates stress-responsive transcription factors, which upregulate p21 expression, resulting in sustained cell-cycle arrest [65,91].

Bioactive peptides have emerged as innovative regulators of CDK inhibitor expression, influencing both transcriptional and post-transcriptional regulatory mechanisms. Peptides derived from dietary and marine sources enhance the expression of p21 and p27 by modulating MAPK and NF-κB signaling pathways, thereby indirectly suppressing CDK activity in colorectal and breast cancer models. Furthermore, alkaloids, including those from plant sources, augment CDK inhibition by activating transcription factors associated with checkpoints and stabilizing p21/p27 proteins [92]. Collectively, these bioactive natural compounds restore the balance between CDKs and their endogenous inhibitors, re-establish checkpoint integrity, and inhibit tumor cell proliferation in breast, colorectal, and pancreatic cancers [63] (Table 2).

**Table 2:** Food-derived natural products targeting cell cycle and apoptosis.

Natural Product Class	Key Compounds	Molecular Targets	Evidence Level	Cancer Types	Representative Clinical Studies	Main Effect	Ref
Alkaloids	Dietary alkaloids	CDKs, apoptotic regulators	Berberine is primarily preclinical; certain medications developed from alkaloids are clinically proven	Pancreatic, colorectal	Early clinical trials with berberine; cancer drugs based on alkaloids have been clinically approved	Cell-cycle arrest, apoptosis	[5]
Polyphenols	Quercetin, Catechins (EGCG)	Cyclin D/E, CDK2/4/6, p21, p27, p53	Limited clinical evidence, mostly in vitro and in vivo	Breast, colorectal, pancreatic, lung	Early dietary and biomarker research involving quercetin and green tea polyphenols	G1/S arrest, apoptosis induction	[11]
Carotenoids	Lycopene, β-carotene	Bax/Bcl-2, ROS signaling	Preclinical, epidemiological, and small-scale clinical research	Prostate, breast, colorectal, lung	Clinical trials with lycopene in patients with prostate cancer	Apoptotic sensitization	[47,55]
Stilbenes	Resveratrol	Cyclin D1, CDKs, p21/p27, p53	Phase I clinical studies combined with preclinical	Colorectal, breast, prostate	Clinical phase I trials showing modulation of Wnt pathway and biomarkers	Checkpoint activation, growth inhibition	[87]
Organosulfur compounds	Sulforaphane, Allicin	Bax/Bcl-2, cytochrome c, caspases	Mostly preclinical	Breast, colorectal, pancreatic, prostate	Pilot human studies with broccoli sprouts	Mitochondrial apoptosis	[67,90]

## 5. Oncogenic Signaling Pathways

### 5.1. PI3K/Akt/mTOR Pathway

The PI3K/Akt and mammalian target of rapamycin (mTOR) signaling pathway constitutes a principal oncogenic axis, consistently regulating essential cellular functions, such as proliferation, survival, metabolism, angiogenesis, autophagy, and therapeutic resistance. This pathway is among the most frequently dysregulated in human cancers, often due to mutations in PI3K catalytic subunits, amplification of receptor tyrosine kinase, or loss of the PTEN tumor suppressor [93].

The activation of this pathway typically occurs when ligands bind to receptor tyrosine kinases, such as the epidermal growth factor receptor (EGFR) or the insulin-like growth factor-1 receptor (IGF-1R). Subsequently, these receptors recruit and activate class I PI3Ks, which catalyze the conversion of phosphatidylinositol-4,5-bisphosphate (PIP2) into phosphatidylinositol-3,4,5-trisphosphate (PIP3). This conversion generates docking sites for Akt and phosphoinositide-dependent kinase-1 3/3 (PDK1) at the plasma membrane [94]. Akt undergoes phosphorylation at Thr308 by PDK1 and at Ser473 by mTOR complex 2 (mTORC2), leading to its complete activation and the initiation of downstream oncogenic signaling [94,95]. Upon activation, Akt phosphorylates various substrates, including BAD, FOXO transcription factors, GSK-3β, and caspase-9, which results in the inhibition of apoptosis, increased glucose metabolism, and facilitation of cell cycle progression.

The mTOR complex 1 (mTORC1) acts as a pivot downstream target of Akt, integrating growth factor signals with the cell’s nutrient and energy status to regulate protein synthesis, lipid production, and ribosomal biogenesis [96]. Akt activates mTORC1 by inhibiting the phosphorylation of the tuberous sclerosis complex (TSC1/2), thereby facilitating the activation of mTOR kinase activity via Rheb-GTP. This activation leads to the phosphorylation of S6 kinase (S6K1) and 4E-binding protein-1 (4E-BP1), which enhances the translation of oncogenic proteins such as cyclin D1, c-Myc, and HIF-1α [97]. Polyphenols, especially flavonoids such as quercetin and catechins, inhibit the enzymatic activity of PI3K by directly competing with ATP for binding at the p110 catalytic subunit [98]. This competition results in reduced PIP3 production, impedes Akt’s recruitment to the membrane, and suppresses downstream mTOR signaling. Consequently, there is a decrease in Akt phosphorylation, which weakens pro-survival and proliferative signaling pathways, thereby promoting apoptosis, and arresting the cell cycle in cancer cells.

Stilbenes, including RSV, inhibit PI3K/Akt signaling by reactivating PTEN expression and function, resulting in enhanced PIP3 dephosphorylation and diminished Akt activation. In addition, they suppress mTORC1 signaling through mechanisms that are both dependent and independent of AMPK.

RSV-induced inhibition of the mTOR signaling pathway results in reduced protein synthesis, angiogenesis, and tumor cell survival, highlighting its significance in metabolic reprogramming and cancer prevention. Curcuminoids, especially curcumin, are among the most extensively studied natural inhibitors of the PI3K/Akt/mTOR pathway. They exert their effects by inhibiting Akt phosphorylation and activating AMP-activated protein kinase (AMPK), a critical negative regulator of mTORC1. Curcumin-induced AMPK activation leads to the inhibition of mTORC1 signaling, decreased phosphorylation of S6K1 and 4E-BP1, enhanced autophagy, and diminished translation of oncogenic proteins. Terpenoids and carotenoids, including lycopene and β-carotene, primarily influence PI3K/Akt/mTOR signaling at the upstream receptor level by inhibiting IGF-1/IGF-1R signaling and reducing oxidative stress-induced activation of growth factor pathways [99].

These compounds inhibit cancer cell proliferation and metabolic adaptation by reducing receptor-mediated PI3K activation, consequently reducing Akt phosphorylation and mTOR signaling. Organosulfur compounds, such as sulforaphane and allicin, effectively suppress the PI3K/Akt/mTOR pathway by inhibiting Akt activation, triggering AMPK-dependent mTOR inhibition, and inducing autophagy [99]. Bioactive peptides and dietary alkaloids, including berberine, inhibit PI3K/Akt/mTOR signaling through AMPK activation, decreased Akt phosphorylation, and mTORC1 activity inhibition, which results in reduced glucose uptake, lipid synthesis, and tumor cell growth [99,100].

### 5.2. MAPK/ERK Signaling Pathways

The mitogen-activated protein kinase (MAPK)/extracellular signal-regulated kinase (ERK) signaling pathways are crucial in mediating cellular responses to growth factors, cytokines, and environmental stresses, thereby influencing proliferation, differentiation, survival, and migration. Hyperactivation of this pathway is a characteristic feature of numerous cancers, often resulting from mutations in RAS, RAF, or continuous receptor tyrosine kinase signaling, which leads to uncontrolled cell growth and survival [101]. The MAPK/ERK signaling pathway acts as a central “decision-making” center within the cell, integrating extracellular growth signals with intracellular programs that regulate proliferation, survival, migration, and differentiation. Under normal physiological conditions, ERK activation facilitates cell cycle progression by inducing cyclin D1 and c-Myc and promotes survival by inhibiting apoptotic processes and enhancing resistance to cellular stress, thereby creating an environment conducive to tumor initiation and progression [101,102].

Polyphenols, including quercetin, catechins, and RSV, exert early-stage control by reducing receptor tyrosine kinase activation and disrupting RAS–RAF interactions, thereby preventing signal amplification from the outset. This initial interference results in reduced phosphorylation of MEK and ERK, subsequently limiting ERK’s movement into the nucleus and its ability to activate transcription factors such as ELK-1, AP-1, and c-Myc. Beyond signal suppression, quercetin directly influences ERK-driven transcriptional activities, resulting in reduced cyclin D1 levels, G1 phase arrest, and increased vulnerability to apoptosis. RSV further enhances this effect by selectively targeting RAS-dependent ERK signaling, thereby reducing tumor growth and the potential for metastasis in various cancer models [103].

Curcumin exemplifies dietary compounds with dual modulatory effects, as it simultaneously inhibits ERK phosphorylation and triggers stress-responsive MAPKs such as JNK and p38 [104]. This alteration in signaling dynamics redirects the cellular response from proliferation to processes such as apoptosis, autophagy, and oxidative stress-induced cell death. A similar reprogramming of signaling pathways is observed with sulforaphane, which inhibits ERK while enhancing the activation of p38 and JNK, thereby disrupting survival signaling and facilitating programmed cell death in cancerous cells. Terpenoids and carotenoids, such as lycopene, also reduce ERK activation by reducing growth factor-mediated signaling and restricting ROS-induced MAPK activation, highlighting the complex relationship between oxidative stress and MAPK dysregulation in cancer [102,104].

Organosulfur compounds, including allicin, as well as dietary alkaloids such as berberine and piperlongumine, modulate ERK inhibition by influencing the regulation of survival proteins. These compounds achieve this by hindering MEK/ERK phosphorylation and reducing ERK activity within the nucleus. Consequently, there is a downregulation of anti-apoptotic proteins, such as Bcl-2, and a suppression of gene transcription that aids tumor cell survival and resistance to chemotherapy [105].

Bioactive natural compounds exert a comprehensive influence on the MAPK/ERK signaling pathway by influencing receptor activation, intracellular kinase cascades, transcription factor involvement, and the expression of survival proteins. These compounds simultaneously inhibit oncogenic ERK signaling and activate stress-induced MAPK pathways, creating a cellular environment that promotes growth arrest and apoptosis in cancer cells, while largely sparing normal tissues. This multi-targeted, modulatory strategy highlights the potential of dietary bioactives as safe and effective chemopreventive agents, especially for long-term cancer prevention strategies aimed at correcting signaling imbalances without causing significant cytotoxicity [104,105].

### 5.3. Wnt/β-Catenin Signaling Pathway

The Wnt/β-catenin signaling pathway is a well-preserved developmental mechanism that plays a crucial role in regulating cell fate determination, proliferation, differentiation, stemness, and tissue homeostasis. In adult tissues, Wnt signaling is precisely regulated and transient. However, in cancer, this pathway becomes persistently active, primarily due to mutations in APC, β-catenin (CTNNB1), or Axin, resulting in the continuous stabilization of β-catenin and subsequent transcriptional activation. This aberrant activation is a hallmark of colorectal cancer and is also frequently observed in hepatocellular, gastric, breast, and pancreatic cancers, where it facilitates tumor initiation, progression, metastatic dissemination, and resistance to chemotherapy [106].

In the canonical Wnt signaling pathway, the interaction of Wnt ligands with Frizzled (FZD) receptors and LRP5/6 co-receptors initiates the recruitment of Disheveled (DVL). This recruitment inhibits the β-catenin destruction complex, which consists of APC, Axin, GSK-3β, and CK1 [107]. Under normal conditions, this complex phosphorylates β-catenin, targeting it for degradation via the ubiquitin-proteasome system. However, upon binding of Wnt ligands, this degradation process is disrupted, leading to the accumulation of β-catenin in the cytoplasm and its subsequent translocation into the nucleus. Within the nucleus, β-catenin associates with TCF/LEF transcription factors and attracts co-activators such as CBP/p300, thereby promoting the expression of oncogenic target genes, including *c-Myc*, *cyclin D1*, *survivin*, *AXIN2*, and matrix metalloproteinases. This activity supports uncontrolled cell proliferation, epithelial–mesenchymal transition (EMT), invasion, and the maintenance of cancer stem cells [108].

Bioactive natural compounds modulate Wnt/β-catenin signaling at various stages, influencing both the initial receptor interactions and the subsequent transcriptional processes [109,110]. Polyphenols, including quercetin, RSV, and various flavonoids, enhance the function of the β-catenin destruction complex by enhancing the phosphorylation of β-catenin through GSK-3β. This process facilitates β-catenin degradation and reduces its nuclear accumulation. Quercetin has been shown to decrease nuclear β-catenin levels, reduce TCF/LEF transcriptional activity, and lower the expression of Wnt target genes, resulting in cell-cycle arrest and apoptosis. Furthermore, RSV interferes with β-catenin–TCF interactions and prevents the recruitment of transcriptional co-activators, thereby inhibiting gene programs associated with proliferation and metastasis.

Curcumin modulates the Wnt signaling pathway through several complementary mechanisms. It stabilizes the destruction complex, inhibits the translocation of β-catenin into the nucleus, and decreases the expression of Wnt ligands and Frizzled receptors. These actions effectively disrupt both autocrine and paracrine Wnt signaling loops in tumors. Furthermore, curcumin inhibits Wnt signaling associated with cancer stem cells, thereby reducing their self-renewal capacity and decreasing the likelihood of tumor recurrence. Compounds such as terpenoids and carotenoids, including lycopene and β-carotene, inhibit LRP6 phosphorylation and prevent Frizzled-mediated signal initiation. Consequently, this results in reduced β-catenin accumulation and reduced expression levels of *c-Myc* and *cyclin D1*.

Organosulfur compounds, including sulforaphane and allicin, attenuate Wnt/β-catenin signaling by facilitating the phosphorylation-dependent degradation and nuclear export of β-catenin. This process impedes the transcription of Wnt target genes and reduces the characteristics of cancer stem cells. Sulforaphane has been shown to inhibit β-catenin signaling in colorectal cancer stem cells, thereby enhancing tumor responsiveness to chemotherapy. Similarly, dietary alkaloids such as berberine reduce β-catenin/TCF transcriptional activity, downregulate Wnt-dependent proliferative genes, and induce cell-cycle arrest [111].

Bioactive natural compounds exert influence across various regulatory nodes within the Wnt/β-catenin pathway. They affect processes ranging from ligand–receptor interactions and the cytoplasmic stabilization of β-catenin to the regulation of nuclear transcription. This multifaceted action helps in restoring β-catenin homeostasis, suppressing oncogenic gene expression, limiting the stemness of cancer cells, and reducing their metastatic potential. Such comprehensive modulation highlights the significant chemopreventive and therapeutic potential of dietary bioactive compounds in cancers driven by aberrant Wnt signaling [110,111].

### 5.4. NF-κB Signaling Pathway

NF-κB signaling pathway functions as a crucial regulator linking chronic inflammation to the initiation, development, and progression of cancer. Under normal circumstances, NF-κB coordinates immune defense and stress responses. However, in oncogenic contexts, persistent activation of NF-κB establishes an inflammatory microenvironment conducive to tumor growth. This environment supports cell survival, uncontrolled cell division, angiogenesis, invasion, immune evasion, and chemotherapy resistance. Constitutive NF-κB activity has been observed in numerous solid and hematological cancers, where it is associated with aggressive tumor behavior, metastatic potential, and poor clinical outcomes [112].

NF-κB comprises a group of transcription factors, predominantly functioning as the p65 (RelA)/p50 heterodimer, which remains sequestered in the cytoplasm of inactive cells due to its interaction with inhibitor of κB (IκB) proteins. In the canonical NF-κB pathway, stimuli such as pro-inflammatory cytokines (e.g., TNF-α and IL-1β), growth factors, carcinogens, and oxidative stress activate the IκB kinase (IKK) complex. This complex consists of the catalytic subunits IKKα and IKKβ, in conjunction with the regulatory subunit NEMO (IKKγ). Upon activation, IKKβ phosphorylates IκBα, leading to its ubiquitination and subsequent degradation by the proteasome. This degradation releases NF-κB, facilitating its translocation into the nucleus. Within the nucleus, NF-κB binds to κB response elements, thereby promoting the transcription of genes encoding anti-apoptotic proteins (Bcl-2, Bcl-xL, and survivin), inflammatory mediators (COX-2, iNOS, TNF-α), cell-cycle regulators (cyclin D1), angiogenic factors (VEGF), and matrix metalloproteinases (MMPs). Collectively, these processes contribute to tumor growth, angiogenesis, invasion, and metastatic dissemination [112,113].

Bioactive natural compounds have the potential to inhibit cancer development induced by inflammation by targeting various regulatory nodes within the NF-κB signaling pathway. Polyphenols, including flavonoids, stilbenes, and curcuminoids, inhibit NF-κB activation at both upstream and downstream levels. Specifically, quercetin and catechins suppress the activation of IKKβ, prevent the phosphorylation and degradation of IκBα, and retain NF-κB in the cytoplasm. This retention results in a reduction in the transcription of genes associated with inflammation and cell survival. RSV further inhibits NF-κB signaling by blocking IKK activity, suppressing p65 phosphorylation, and impairing NF-κB’s DNA-binding activity. Consequently, there is decreased expression of COX-2, iNOS, and anti-apoptotic proteins in cancer cells. Among dietary NF-κB inhibitors, curcumin is notably effective, as it directly inhibits IKK activity, stabilizes IκBα, and prevents the nuclear translocation and transcriptional activity of p65. This action reduces inflammation, promotes apoptosis, and inhibits tumor growth [113].

Terpenoids and carotenoids, including lycopene and β-carotene, primarily influence NF-κB signaling through antioxidant-dependent and redox-related mechanisms. These compounds reduce intracellular ROS, consequently attenuating ROS-induced activation of IKK and NF-κB, which curtails chronic inflammatory signaling associated with tumor progression. Lycopene has been shown to inhibit NF-κB-dependent transcription of VEGF and MMPs, resulting in decreased angiogenesis and metastatic potential. Organosulfur compounds, including sulforaphane, inhibit NF-κB signaling by covalently modifying essential cysteine residues in IKKβ, leading to reduced IKK activity, stabilization of IκBα, and inhibition of NF-κB nuclear translocation. In addition, sulforaphane disrupts NF-κB-mediated cancer stem cell survival and enhances susceptibility to apoptosis, underscoring its relevance in early cancer prevention [114].

Dietary alkaloids and bioactive peptides play a significant role in the inhibition of NF-κB. Specifically, alkaloids such as berberine prevent NF-κB activation by inhibiting IKK phosphorylation and the nuclear translocation of p65. This process leads to the downregulation of inflammatory cytokines and anti-apoptotic genes, while promoting apoptosis through the activation of caspases. Furthermore, certain peptides have been shown to suppress NF-κB-dependent signaling, thereby contributing to the reduction in tumor cell proliferation and inflammation associated tumor promotion [114,115].

### 5.5. STAT3 Signaling Pathway

**Figure 3:**
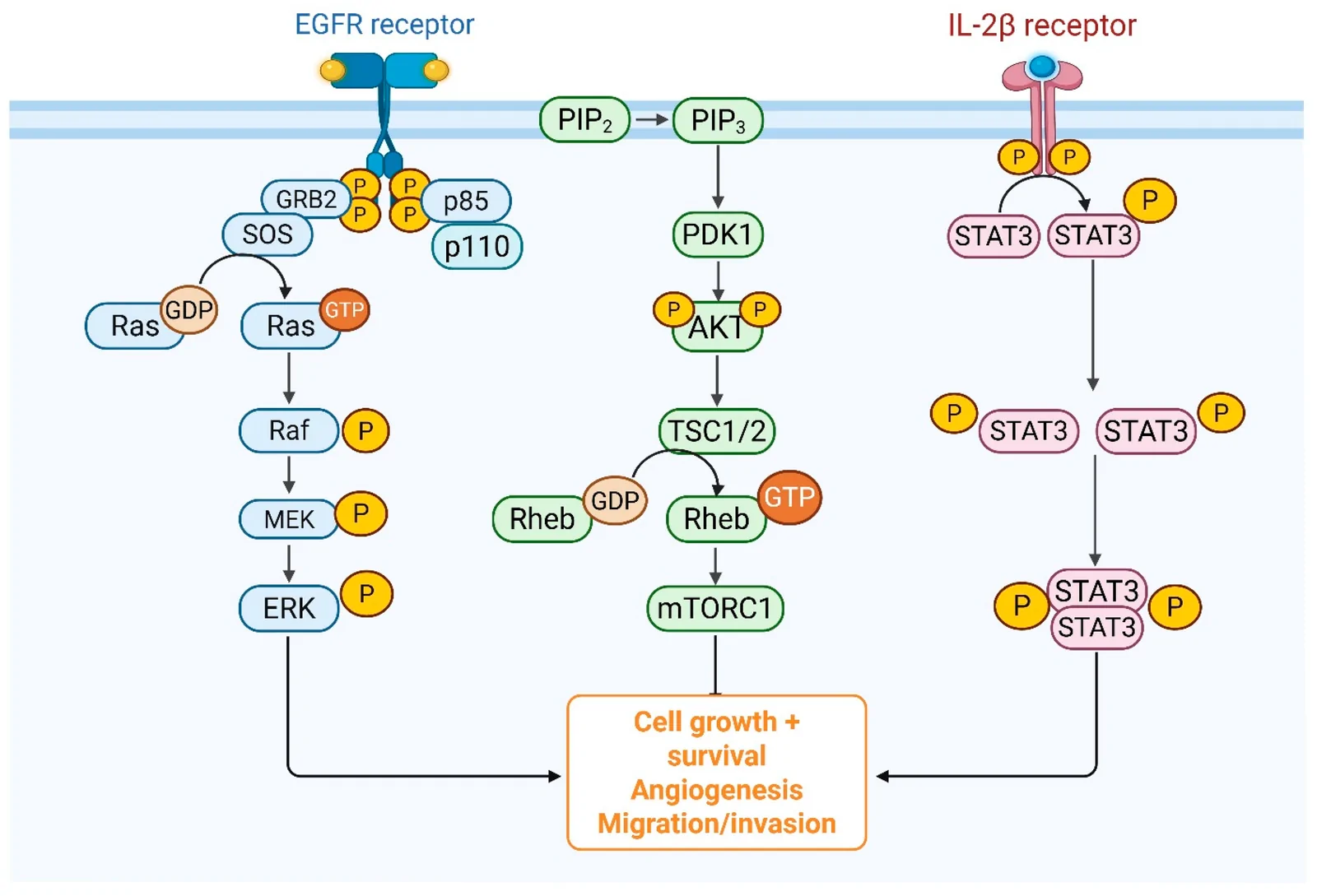
EGF and IL-2 Signaling Pathways.

Signal transducer and activator of transcription 3 (STAT3) is a latent cytoplasmic transcription factor located in the cytoplasm, crucial for integrating signals from cytokines, growth factors, and oncogenic kinases. Under physiological conditions, STAT3 is typically activated rapidly and transiently, facilitating the regulated control of cell growth, differentiation, immune responses, and tissue repair. In contrast, in oncogenic contexts, STAT3 becomes constitutively active due to continuous stimulation by inflammatory cytokines such as interleukin-6 (IL-6), hyperactivation of growth factor receptors, or aberrant activity of upstream kinases, including Janus kinases (JAKs), Src family kinases, and receptor tyrosine kinases (Figure 3). Continuous STAT3 signaling is a hallmark of various solid and hematological malignancies, contributing to tumor initiation, progression, immune evasion, angiogenesis, metastasis, and chemotherapy resistance [116].

Canonical activation of STAT3 is initiated when cytokines or growth factors bind to their specific receptors, leading to the activation of JAK1/2, Src, or EGFR associated with the receptor. These kinases subsequently phosphorylate STAT3 at the conserved Tyr705 residue, triggering STAT3 to dimerize through reciprocal interactions of the SH2 domain. Upon dimerization, STAT3 translocates into the nucleus, where it binds to STAT-responsive elements in promoter regions and recruits transcriptional co-activators to enhance the expression of oncogenic target genes. These genes include anti-apoptotic proteins (e.g., Bcl-2, Bcl-xL, and survivin), cell-cycle regulators (e.g., cyclin D1 and c-Myc), angiogenic factors (e.g., VEGF), immune checkpoint molecules (e.g., PD-L1), invasion-associated enzymes (e.g., MMP-2 and MMP-9), and inflammatory cytokines such as IL-6. This mechanism establishes a self-sustaining feed-forward loop that maintains STAT3 activation and supports tumor progression [116,117].

Bioactive natural compounds exhibit significant chemopreventive effects by targeting STAT3 signaling at various regulatory checkpoints. Polyphenols, including flavonoids, stilbenes, and curcuminoids, primarily inhibit STAT3 activation by obstructing upstream kinases, thereby preventing Tyr705 phosphorylation and the subsequent formation of STAT3 dimers. Quercetin has been shown to inhibit IL-6-induced STAT3 phosphorylation, reduce the nuclear accumulation of STAT3, and inhibit the transcription of STAT3-dependent anti-apoptotic genes, leading to enhanced apoptosis and growth inhibition. Similarly, RSV interferes with STAT3 signaling by preventing JAK/STAT phosphorylation, impeding STAT3 nuclear translocation, and downregulating the expression of STAT3 target genes associated with cell proliferation and angiogenesis [118].

Curcumin is acknowledged as a potent natural inhibitor of STAT3, exerting its effects through several complementary mechanisms. These mechanisms encompass the direct inhibition of JAK kinase activity, the reduction in STAT3 phosphorylation, and the prevention of STAT3′s binding to DNA. The inhibition of STAT3 by curcumin results in the downregulation of survivin, Bcl-2, and VEGF, induces caspase-dependent apoptosis, and hinders tumor growth and metastasis across various cancer models. Notably, curcumin also interferes with the positive feedback loop between STAT3 and IL-6 signaling, thereby diminishing inflammation-driven tumor promotion and the ongoing activation of STAT3 [118,119].

Terpenoids and carotenoids indirectly modulate STAT3 signaling by suppressing inflammation and oxidative stress pathways, which are responsible for persistent STAT3 activation. Lycopene inhibits IL-6-induced STAT3 phosphorylation and reduces the transcription of STAT3-dependent genes associated with angiogenesis and proliferation, thereby contributing to the prevention of tumor growth. Organosulfur compounds, including sulforaphane, prevent STAT3 activation by inhibiting JAK2 phosphorylation and causing epigenetic suppression of STAT3-regulated genes, while also promoting apoptosis and reducing cancer stem cell survival. Furthermore, sulforaphane weakens STAT3-driven immunosuppressive signaling within the tumor microenvironment, thereby enhancing anti-tumor immune responses [118,120].

Dietary alkaloids, such as berberine, inhibit STAT3 signaling by preventing the activation of upstream kinases, thereby blocking STAT3 phosphorylation and nuclear translocation, which subsequently reduces the expression of STAT3- dependent oncogenic genes. This inhibition leads to decreased cell proliferation, increased apoptosis, and decreased metastasis. In addition, bioactive peptides from food proteins have been shown to attenuate STAT3 signaling, aiding in the reduction in inflammation-associated cancer progression and tumor growth [116,117].

## 6. Epigenetic Regulation by Food-Derived Compounds

### 6.1. Inhibition of DNMTs and HDACs

Epigenetic alterations, including aberrant DNA methylation and histone deacetylation, are integral to the initiation and progression of cancer, as they consistently silence tumor suppressor genes without altering the DNA sequence. In malignancies such as breast, colorectal, and pancreatic cancers, the overexpression of DNA methyltransferases (DNMTs) and histone deacetylases (HDACs) results in the hypermethylation of promoter regions and a more condensed chromatin structure, thereby suppressing genes responsible for cell cycle arrest, apoptosis, and DNA repair. Bioactive natural compounds, especially curcuminoids, serve as significant epigenetic modulators by targeting both DNMTs and HDACs [5,121].

Polyphenols, including quercetin, catechins, and curcumin, inhibit DNMT1 activity through direct interaction with its catalytic domain. This interaction resulted in extensive DNA hypomethylation and the reactivation of previously silenced genes. In breast cancer, curcumin-mediated DNMT inhibition restores the expression of tumor suppressor genes regulated by estrogen receptor. In colorectal cancer, curcumin reverses the aberrant methylation of genes associated with Wnt signaling and apoptosis. Pancreatic cancer, characterized by significant epigenetic silencing, responds to curcumin through reduced expression of DNMT and HDAC, leading to the reprogramming of malignant epigenetic profiles [94,122]. RSV functions by inhibiting DNMTs and HDACs, facilitating chromatin relaxation and the reactivation of tumor suppressor genes [123]. In addition, the regulation of cancer-associated microRNAs aids in the suppression of EMT, cell proliferation, and metastasis.

Carotenoids, such as lycopene and β-carotene, play an indirect role in epigenetic regulation by reducing oxidative stress and inflammatory signaling. These processes are linked to elevated DNMT and HDAC activity. By inhibiting these epigenetic repressors, bioactive natural compounds enhance chromatin accessibility and transcriptional activity of genes that confer protection against cancer. This underscores their significance in cancer prevention strategies.

Sulforaphane, a prominent isothiocyanate found in cruciferous vegetables such as broccoli, effectively inhibits DNMT1 and class I HDACs. Mechanistically, sulforaphane covalently modifies cysteine residues within the catalytic domains of these enzymes, resulting in decreased methylation at tumor suppressor gene promoters. In breast cancer, the inhibition of DNMT by sulforaphane restores estrogen receptor-regulated tumor suppressor genes, thereby enhancing transcriptional activity and reestablishing growth control. In colorectal cancer, sulforaphane counteracts aberrant methylation of Wnt pathway inhibitors and apoptosis-regulating genes, thus suppressing oncogenic signaling. A reduction in sulforaphane decreases the expression of DNMT and HDAC, which reprograms the malignant epigenetic landscapes and reactivates growth-suppressive genes in pancreatic cancer, leading to extensive epigenetic silencing [63].

Allicin, a sulfur-containing molecule found in garlic, has been shown to exert an indirect influence on epigenetic regulation. In models of breast and colorectal cancer, allicin enhances the transcription of tumor suppressor genes and restores chromatin accessibility by modifying the intracellular redox balance and reducing oxidative stress-driven DNMT and HDAC. Furthermore, allicin consistently inhibits HDAC activity and mitigates oxidative DNA damage in pancreatic cancer, thereby supporting the epigenetic regulation of cell cycle and apoptosis-related genes [65].

Bioactive peptides and certain alkaloids influence epigenetic regulation by modulating signaling pathways that affect DNMT and HDAC activities. The activation of phosphatase pathways by peptides and the suppression of MAPK/NF-κB by alkaloids may diminish epigenetic silencing in cancer cells, thereby promoting the re-expression of tumor suppressor genes such as *p16* and *RASSF1A*. In combination, these bioactive agents achieve epigenetic equilibrium through the regulation of DNMT, HDAC, and chromatin modification, which results in the activation of tumor suppressors and cancer prevention strategies for breast, colorectal, and pancreatic tumors [81].

### 6.2. Regulation of Cancer-Related microRNAs

MicroRNAs (miRNAs) are short non-coding RNAs that regulate gene expression at the post-transcription level and are integral to cancer development. Dysregulated miRNA expression contributes to oncogenesis by either suppressing tumor suppressor genes or enhancing oncogenic signaling pathways. In breast, colorectal, and pancreatic cancers, there is a frequent overexpression of oncogenic miRNAs, such as miR-21, alongside a downregulation of tumor suppressor miRNAs, such as miR-34a and miR-200 family. Bioactive natural compounds exert miRNA expression through epigenetic mechanisms, thereby affecting cancer-related signaling pathways [124].

Polyphenols modulate miRNA expression by affecting both transcriptional regulation and epigenetic mechanisms. In breast cancer, curcumin downregulates miR-21 levels, resulting in increased PTEN expression and suppression of the PI3K/Akt signaling pathway. In colorectal cancer, curcumin induces miR-34a expression, thereby enhancing p53-mediated apoptosis. In pancreatic cancer, curcumin restores miR-200 expression, which inhibits epithelial–mesenchymal transition (EMT) and metastatic progression [125,126,127]. Carotenoids influence miRNA regulation by modulating oxidative stress and inflammatory pathways, subsequently impacting miRNA biogenesis. Lycopene has been shown to regulate miRNAs associated with cell cycle control and apoptosis, reinforcing its chemopreventive role [124]. Sulforaphane, an extensively studied organosulfur compound from cruciferous vegetables, significantly regulates cancer-associated miRNAs by modulating epigenetic enzymes and transcription factors. In breast cancer cells, sulforaphane downregulates the oncogenic miR-21, leading to PTEN re-expression of and PI3K/Akt signaling pathway suppression, thereby inhibiting cell proliferation and survival. In colorectal cancer, sulforaphane induces tumor-suppressive miR-34a expression via p53 activation, promoting apoptosis and cell cycle arrest. In pancreatic cancer, sulforaphane restores miR-200 family expression, suppressesing EMT regulators such as ZEB1 and Snail, thereby reducing invasion and metastatic potential [124]. Allicin, a compound derived from garlic, influences miRNA regulation by modulating intracellular redox balance and inflammatory signaling pathways, affecting miRNA biogenesis. In breast and colorectal cancer models, allicin suppresses NF-κB-dependent oncogenic miRNAs, such as miR-21 and miR-155, while enhancing tumor-suppressive miRNAs involved in apoptosis and growth inhibition. In pancreatic cancer, allicin-mediated miRNA modulation reduces EMT-associated miRNAs and sensitizes tumor cells to apoptotic signals [65].

Bioactive peptides and alkaloids modulate miRNA expression by targeting signaling pathways, such as MAPK, STAT3, and NF-κB, which are responsible for miRNA transcription regulation. Alkaloids, such as berberine, have been shown to upregulate miR-34a while downregulating miR-21 in various cancer types, thereby reinforcing tumor suppressor networks. In summary, organosulfur compounds, bioactive peptides, and alkaloids exert precise epigenetic control over miRNA expression, thereby restoring the balance between oncogenic and tumor-suppressive miRNAs. This modulation contributes to the prevention and suppression of breast, colorectal, and pancreatic cancers [128].

### 6.3. Histone Acetylation and Chromatin Remodeling

**Figure 4:**
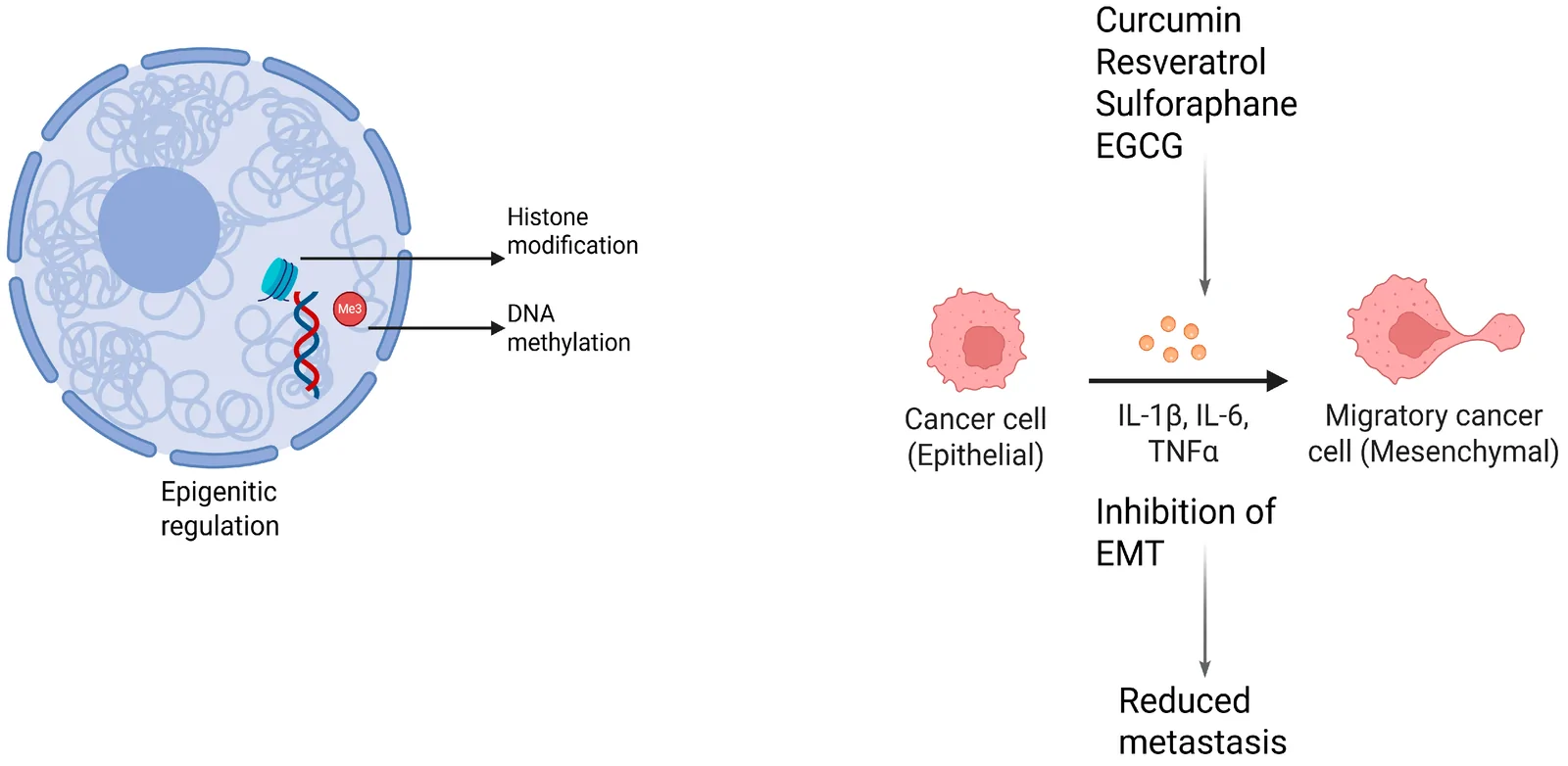
Epigenetic regulation and anti-metastatic effects.

Histone modifications play a critical role in the regulation of chromatin structure and gene expression. In the context of cancer, increased HDAC activity results in histone hypoacetylation, which causes chromatin compaction and the transcriptional repression of tumor suppressor genes. In breast, colorectal, and pancreatic cancers, aberrant histone deacetylation contributes to tumor progression and therapy resistance. Bioactive natural compounds, particularly curcuminoids, have been shown to modulate histone acetylation and chromatin remodeling [129] (Figure 4).

Polyphenols inhibit HDAC activity, leading to increased acetylation of histones H3 and H4. This alteration of chromatin structure facilitates the reactivation of genes associated with cell cycle arrest, apoptosis, and DNA repair. In the context of breast cancer, curcumin-induced histone acetylation augments the expression of p21 and other checkpoint regulators. In colorectal cancer, curcumin-mediated chromatin remodeling suppresses oncogenic transcriptional programs. Pancreatic cancer cells demonstrate increased sensitivity to curcumin-mediated chromatin relaxation, resulting in growth inhibition and apoptosis [130].

Carotenoids play a role in chromatin remodeling by mitigating epigenetic repression caused by oxidative stress. These bioactive natural compounds target histone modifications and chromatin structure to restore epigenetic equilibrium and inhibit malignant phenotypes [124,131].

Sulforaphane, a potent organosulfur compound derived from cruciferous vegetables, is one of the most thoroughly characterized natural HDAC inhibitors identified to date. It directly inhibits class I and II HDACs, leading to increased acetylation of histones H3 and H4 at the promoter regions of tumor suppressor genes. In breast cancer models, sulforaphane-induced histone hyperacetylation reactivates genes such as *p21*, *Bax*, and *BRCA1*, resulting in cell cycle arrest and apoptosis. In colorectal cancer, sulforaphane-mediated chromatin relaxation restores the expression of Wnt pathway antagonists and pro-apoptotic genes, thereby suppressing β–catenin-driven transcriptional programs. Pancreatic cancer cells, which exhibit significant epigenetic repression, show increased sensitivity to sulforaphane-induced chromatin remodeling, leading to growth inhibition and enhanced apoptotic signaling [82,122]. Allicin, another organosulfur compound from garlic, contributes to histone acetylation indirectly by modulating redox status and inflammatory signaling pathways that regulate HDAC expression and activity. In breast and colorectal cancer cells, allicin suppresses NF-κB-dependent HDAC upregulation, partially restoring histone acetylation and re-expressing growth-inhibitory genes. In pancreatic cancer, allicin-mediated chromatin relaxation sensitizes tumor cells to apoptosis and limits inflammatory epigenetic reprogramming within the tumor microenvironment [68].

Bioactive peptides and alkaloids consistently regulate chromatin remodeling by targeting signaling pathways that regulate the expression of epigenetic enzymes. Certain dietary peptides have been shown to enhance HAT activity, thereby promoting histone acetylation and facilitating the transcription of tumor suppressor genes. Alkaloids, such as berberine and related compounds, exhibit HDAC-inhibitory activity, which promotes histone acetylation at the promoters of genes involved in cell cycle arrest and apoptosis. In breast, colorectal, and pancreatic cancer models, these compounds contribute to epigenetic reprogramming that counteracts malignant phenotypes [81].

## 7. Inhibition of Angiogenesis and Metastasis

### 7.1. VEGF and VEGFR

VEGF signaling constitutes a fundamental mechanism in tumor angiogenesis, facilitating endothelial cell proliferation, migration, and the development of new blood vessels. The overexpression of VEGF and VEGFR-2 is frequently observed in breast, colorectal, and pancreatic cancers, correlating with poor prognosis, increased tumor vascularity, and enhanced metastatic potential. Hypoxia-inducible factor-1α (HIF-1α) plays a crucial role in the transcription of VEGF under hypoxic tumor conditions. Bioactive natural compounds have been shown to effectively disrupt this angiogenic pathway [130,132].

Polyphenols have been shown to suppress VEGF expression by inhibiting the stabilization and transcriptional activity of HIF-1α. In breast cancer models, curcumin reduces VEGF secretion and inhibits VEGFR-2 phosphorylation, thereby preventing endothelial cell proliferation and tube formation. In colorectal cancer, curcumin downregulates VEGF by suppressing NF-κB and COX-2 signaling pathways, leading to impaired angiogenic responses. In pancreatic cancer, characterized by intense hypoxia and angiogenic signaling, curcumin leads to reduced VEGF expression and impaired tumor vascularization [130].

Previous research has established that curcumin inhibits both the proliferation of vascular endothelial cells in vitro and the formation of capillary tubes in vivo. Curcumin has been shown to downregulate the mRNA and protein expression of VEGF induced by hypoxia, thereby suppressing angiogenesis stimulated by hypoxia. Huang et al. investigated the impact of curcumin on endothelial cell migration, attachment, and tube formation on Matrigel [133]. The findings indicated that curcumin treatment led to a dose-dependent inhibition of tube formation and metalloproteinase activities. In addition, it inhibited angiogenesis in a subcutaneous Matrigel plug model in mice.

Lycopene demonstrates anti-angiogenic properties by inhibiting the induction of VEGF mediated by IGF-1, with significant effects observed in breast and colorectal cancers. β-Carotene aids in this process by mitigating oxidative stress, which would otherwise increase HIF-1α-dependent VEGF expression. By collectively inhibiting VEGF/VEGFR signaling, these bioactive natural compounds effectively deprive tumors of their blood supply, thereby restricting growth and metastatic dissemination [35,134].

Organosulfur compounds, particularly sulforaphane, have been shown to effectively suppress VEGF-driven angiogenesis through comprehensive molecular regulation. Sulforaphane inhibits VEGF expression by preventing the stabilization and nuclear accumulation of HIF-1α under hypoxic conditions. In breast cancer models, sulforaphane decreases VEGF secretion from tumor cells and attenuates VEGFR-2 phosphorylation in endothelial cells, thereby impairing endothelial proliferation and tube formation. In colorectal cancer, sulforaphane suppresses VEGF transcription by inhibiting the NF-κB and STAT3 signaling pathways, both of which contribute to inflammation- and hypoxia-induced angiogenesis. In pancreatic cancer, where hypoxia and KRAS-driven signaling strongly induce VEGF expression, sulforaphane disrupts HIF-1α–VEGF signaling, resulting in reduced tumor vascularization and impaired tumor growth [82]. Allicin further contributes to anti-angiogenic activity by modulating redox-sensitive angiogenic signaling. By altering the intracellular thiol balance and suppressing oxidative stress-induced HIF-1α activation, allicin reduces VEGF expression in breast and colorectal cancer cells [135,136]. In pancreatic cancer, allicin interferes with VEGF-mediated endothelial cell migration and vessel formation, thereby limiting the development of functional tumor vasculature. These effects weaken the angiogenic support system required for rapid tumor expansion and metastatic spread [65].

Bioactive peptides exhibit anti-angiogenic effects by targeting VEGF signaling directly or indirectly. A range of dietary and marine-derived peptides inhibit VEGF-induced proliferation and migration of endothelial cells, consistently suppressing VEGFR-2 activation in colorectal and breast cancer models. Alkaloids further enhance these effects by blocking VEGF transcription and disrupting downstream VEGFR-mediated signaling pathways, such as the PI3K/Akt and MAPK pathways. By collectively inhibiting VEGF/VEGFR signaling, organosulfur compounds, bioactive peptides, and alkaloids effectively deprive tumors of their blood supply. This action limits tumor growth, angiogenic expansion, and metastatic dissemination in breast, colorectal, and pancreatic cancers [81].

### 7.2. E-Cadherin and EMT Markers

The EMT, characterized by the loss of epithelial characteristics and the acquisition of mesenchymal traits, is a pivotal process in cancer metastasis. This transition involves the downregulation of E-cadherin, a key cell–cell adhesion molecule, and the upregulation of EMT markers such as N-cadherin, vimentin, Snail, and Twist, which collectively enhance tumor cell motility and invasiveness. EMT is particularly active in triple-negative breast cancer, advanced colorectal cancer, and pancreatic cancer, thereby contributing to their aggressive metastatic behavior [94,137]. Curcumin has shown the ability to reverse EMT by restoring E-cadherin expression and suppressing mesenchymal markers. In breast cancer, curcumin inhibits EMT by suppressing TGF-β and NF-κB signaling pathways, which leads to reduced invasion and migration. In colorectal cancer, curcumin blocks β-catenin nuclear translocation, thereby stabilizing E-cadherin-mediated adhesion. In pancreatic cancer cells, curcumin treatment results in partial EMT reversal, which decreases metastatic potential [76]. Furthermore, carotenoids contribute to EMT inhibition by reducing oxidative stress and inflammatory signaling that activate EMT. Lycopene has been shown to suppress EMT markers and inhibit migration in breast and colorectal cancer cells. By preserving epithelial integrity and suppressing EMT programs, bioactive natural compounds limit metastatic dissemination.

Organosulfur compounds, especially sulforaphane, have shown efficacy in reversing EMT by restoring epithelial phenotypes and suppressing mesenchymal signaling. Sulforaphane enhances the expression of E-cadherin while concurrently downregulating N-cadherin, vimentin, and EMT-inducing transcription factors through the inhibition of TGF-β, NF-κB, and Wnt/β-catenin signaling pathways. In breast cancer models, sulforaphane suppresses EMT and reduces the migratory and invasive capacities of highly aggressive cells. In colorectal cancer, it inhibits β-catenin nuclear translocation, stabilizes E-cadherin-mediated cell adhesion, and limits EMT-driven invasion. Pancreatic cancer cells, which frequently exist in a partial EMT state, exhibit EMT reversal following sulforaphane treatment, resulting in reduced motility and metastatic potential [3,56,85]. Allicin also contributes to EMT suppression by modulating redox-sensitive signaling pathways involved in EMT activation. By reducing oxidative stress and inhibiting NF-κB and TGF-β-dependent transcriptional programs, allicin restores E-cadherin expression and suppresses mesenchymal marker expression in breast and colorectal cancer cells. In pancreatic cancer, allicin disrupts EMT-associated cytoskeletal remodeling, thereby reducing invasive and migratory behavior [138] Bioactive peptides are emerging as anti-EMT agents by regulating key signaling cascades involved in cell plasticity. Several diet-derived peptides suppress EMT by downregulating Snail and Twist expression and restoring epithelial markers, particularly in colorectal and breast cancer models. Alkaloids further reinforce EMT inhibition by targeting transcriptional regulators of EMT and disrupting signaling pathways such as PI3K/Akt and MAPK that sustain mesenchymal phenotypes. Through the coordinated restoration of E-cadherin expression and suppression of EMT-associated markers, organosulfur compounds, bioactive peptides, and alkaloids effectively limit tumor cell invasion, metastatic dissemination, and therapy resistance across breast, colorectal, and pancreatic cancers.

## 8. Oxidative Stress and Inflammation-Mediated Cancer Prevention

### 8.1. ROS Modulation

Reactive oxygen species (ROS) play a dual role in cancer biology. While physiological levels of ROS are essential for normal cellular signaling, excessive ROS production leads to DNA damage, genomic instability, and the activation of oncogenic pathways, thereby facilitating tumor initiation and progression. In breast, colorectal, and pancreatic cancers, chronic oxidative stress contributes to mutagenesis, sustained proliferative signaling, and resistance to apoptosis. Elevated ROS levels activate redox-sensitive transcription factors such as NF-κB and AP-1, thereby linking oxidative stress to inflammation-driven carcinogenesis [139]. 

Bioactive natural compounds, particularly curcuminoids and carotenoid terpenoids such as lycopene and β-carotene, exhibit significant antioxidant properties by scavenging free radicals and restoring intracellular redox balance. Curcumin directly neutralizes ROS and enhances the expression of endogenous antioxidant enzymes, including superoxide dismutase (SOD), catalase, and glutathione peroxidase. In breast cancer models, curcumin mitigates ROS-mediated DNA damage and inhibits redox-driven proliferation. In colorectal cancer, oxidative stress resulting from chronic inflammation and gut dysbiosis is reduced by curcumin through the suppression of lipid peroxidation and oxidative DNA lesions. Pancreatic cancer cells, which experience high intrinsic oxidative stress due to oncogenic KRAS activation, are particularly susceptible to redox modulation by curcumin, leading to growth inhibition and apoptosis [140].

Rahman et al. conducted a study on the effects of curcumin on human non-small cell lung cancer NCI-H460 cells [141]. Their findings demonstrated that curcumin induces apoptosis in NCI-H460 cells in a dose-dependent manner. Specifically, curcumin treatment resulted in the upregulation of BAX and BAD, while BCL-2, BCL-XL, and XIAP were downregulated. In addition, exposure to curcumin led to an increase in ROS, intracellular Ca^2+^, and endoplasmic reticulum (ER) stress in NCI-H460 cells. It is suggested that the generation of reactive oxygen intermediates facilitates the release of cytochrome *c*, thereby inducing tumor cell apoptosis following curcumin treatment. Furthermore, curcumin is capable of inducing mitochondrial abnormalities, promoting p53-dependent apoptosis, and activating caspase-8 and caspase-3 [141].

Lycopene and β-carotene function as lipid-soluble antioxidants, protecting cellular membranes from oxidative damage. Lycopene is particularly effective in quenching singlet oxygen and has always been associated with reduced oxidative stress markers in colorectal and breast cancer tissues. By modulating ROS levels, these natural compounds prevent oxidative DNA damage and disrupt redox-dependent oncogenic signaling, thereby contributing to cancer prevention [142].

Organosulfur compounds, particularly sulforaphane, serve as potent modulators of intracellular redox homeostasis. Sulforaphane exhibits a dual antioxidant–pro-oxidant effect that is dependent on the cellular environment, effectively protecting normal cells while sensitizing cancer cells to oxidative stress-induced apoptosis. In the context of breast cancer, sulforaphane mitigates excessive ROS accumulation by inducing phase II detoxifying and antioxidant enzymes, thereby limiting oxidative DNA damage and suppressing redox-driven proliferation. In colorectal cancer, where oxidative stress is often exacerbated by chronic inflammation and gut-derived reactive metabolites, sulforaphane reduces ROS levels and inhibits lipid peroxidation, thereby decreasing mutagenic pressure. Pancreatic cancer cells, characterized by high intrinsic ROS due to oncogenic KRAS signaling and metabolic reprogramming, are particularly sensitive to sulforaphane-mediated redox modulation, leading to growth inhibition and apoptosis [139]. Allicin also plays a significant role in ROS modulation through its activity as a reactive sulfur species. Allicin transiently increases intracellular ROS in cancer cells beyond a tolerable threshold, inducing oxidative stress-mediated apoptosis, while simultaneously enhancing antioxidant defenses in non-malignant cells. In breast and colorectal cancer models, allicin-induced ROS accumulation disrupts mitochondrial membrane potential and activates intrinsic apoptotic signaling. In pancreatic cancer, allicin exacerbates oxidative stress in cells already burdened with ROS, leading to mitochondrial dysfunction and caspase activation. This selective redox imbalance renders allicin particularly effective in targeting cancer cells with high oxidative vulnerability [65,68].

Bioactive peptides play a crucial role in redox regulation by enhancing endogenous antioxidant systems and suppressing enzymes responsible for ROS. Peptides derived from dietary and marine sources enhance the activity of antioxidant enzymes, such as superoxide dismutase and glutathione peroxidase, thereby restoring redox equilibrium in colorectal and breast cancer cells. Alkaloids complement these effects by modulating redox-sensitive signaling pathways and inhibiting ROS-induced NF-κB activation. Through the coordinated regulation of ROS production and antioxidant defenses, organosulfur compounds, bioactive peptides, and alkaloids prevent oxidative DNA damage, suppress redox-dependent oncogenic signaling, and restrict cancer progression in breast, colorectal, and pancreatic cancers [81].

### 8.2. Inhibition of COX-2, iNOS, and Pro-Inflammatory Cytokines

Chronic inflammation is acknowledged as a contributing factor in both the development and progression of cancer. Inflammatory mediators, including cyclooxygenase-2 (COX-2), inducible nitric oxide synthase (iNOS), and pro-inflammatory cytokines (e.g., TNF-α, IL-6, and IL-1β), are often overexpressed in breast, colorectal, and pancreatic cancers. These mediators facilitate tumor growth by promoting proliferation, angiogenesis, invasion, and immune evasion [143,144].

Curcumin serves as a potent anti-inflammatory agent, consistently suppressing the expression of COX-2 and iNOS by the inhibition of NF-κB and STAT3 signaling pathways. In colorectal cancer, the overexpression of COX-2 is closely associated with inflammation-driven tumorigenesis, and curcumin has been shown to consistently reduce prostaglandin E2 production and tumor burden. In breast cancer, curcumin downregulates inflammatory cytokines that facilitate tumor growth and metastasis. Pancreatic cancer, which is characterized by a highly inflammatory tumor microenvironment, exhibits reduced cytokine secretion and suppression of inflammation-mediated tumor progression [113,116]. Wang et al. investigated the effect of curcumin on deoxycholic acid (DCA)-induced cell proliferation in the human HT-29 colon cancer cell line, focusing on its underlying molecular mechanisms [145]. Their findings indicated that curcumin treatment inhibited cell proliferation by regulating COX-2 mRNA transcription, COX-2 protein expression, and PGE2 synthesis induced by DCA in the HT-29 cell line [145]. In addition, RSV was found to dose-dependently inhibit both COX-2 induction and PGE2 production in bFGF-stimulated fibroblasts [116].

Carotenoids augment the anti-inflammatory effects by mitigating the activation of the inflammatory pathways triggered by oxidative stress. Lycopene has been shown to inhibit the production of IL-6 and TNF-α in colorectal and breast cancer models. By targeting inflammatory mediators, bioactive natural compounds interfere with the pro-tumorigenic inflammatory environment, which is essential for cancer development [2,54,146].

Organosulfur compounds, particularly sulforaphane, exhibit significant anti-inflammatory activity by targeting various inflammatory signaling pathways. Sulforaphane suppresses the expression of COX-2 and iNOS through the inhibition of NF-κB and STAT3 activation, which are crucial transcription factors involved in the expression of inflammatory genes in cancer cells. In colorectal cancer models, sulforaphane significantly reduces prostaglandin E2 synthesis by downregulating COX-2, thereby mitigating inflammation associated with tumor initiation and progression. In breast cancer, sulforaphane attenuates the production of IL-6 and TNF-α, disrupting cytokine-mediated interactions between tumor cells and the surrounding stromal and immune cells (Figure 4). Pancreatic cancer, characterized by a highly desmoplastic and inflammatory tumor microenvironment, exhibits significant sensitivity to sulforaphane-induced suppression of cytokine secretion, resulting in reduced inflammatory signaling and inhibition of tumor growth [63]. Allicin also plays a role in controlling inflammation by modulating redox-sensitive inflammatory pathways. As a reactive sulfur species, allicin interferes with NF-κB nuclear translocation and reduces iNOS expression, leading to decreased nitric oxide production in cancer cells. In breast and colorectal cancer models, allicin suppresses TNF-α- and IL-1β-mediated inflammatory signaling, thereby inhibiting inflammation-driven proliferation and invasion. In pancreatic cancer, allicin reduces cytokine-induced activation of survival pathways, weakening the inflammatory support that sustains tumor progression [67].

Bioactive peptides and alkaloids exert enhanced anti-inflammatory effects by specifically targeting cytokine signaling and inflammatory enzymes. A range of dietary and marine-derived peptides inhibit the expression of COX-2 and iNOS through modulation of MAPK and NF-κB signaling pathways, with efficacy observed in colorectal and breast cancer models. In addition, alkaloids suppress the production of pro-inflammatory cytokines and disrupt inflammatory feedback loops within the tumor microenvironment. By coordinating the inhibition of COX-2, iNOS, and pro-inflammatory cytokines, organosulfur compounds, bioactive peptides, and alkaloids effectively dismantle the pro-tumorigenic inflammatory environment. This coordinated action is crucial in the prevention and suppression of cancer, particularly in breast, colorectal, and pancreatic cancers [81].

## 9. Omics-Based Approaches

### 9.1. Transcriptomics

**Figure 5:**
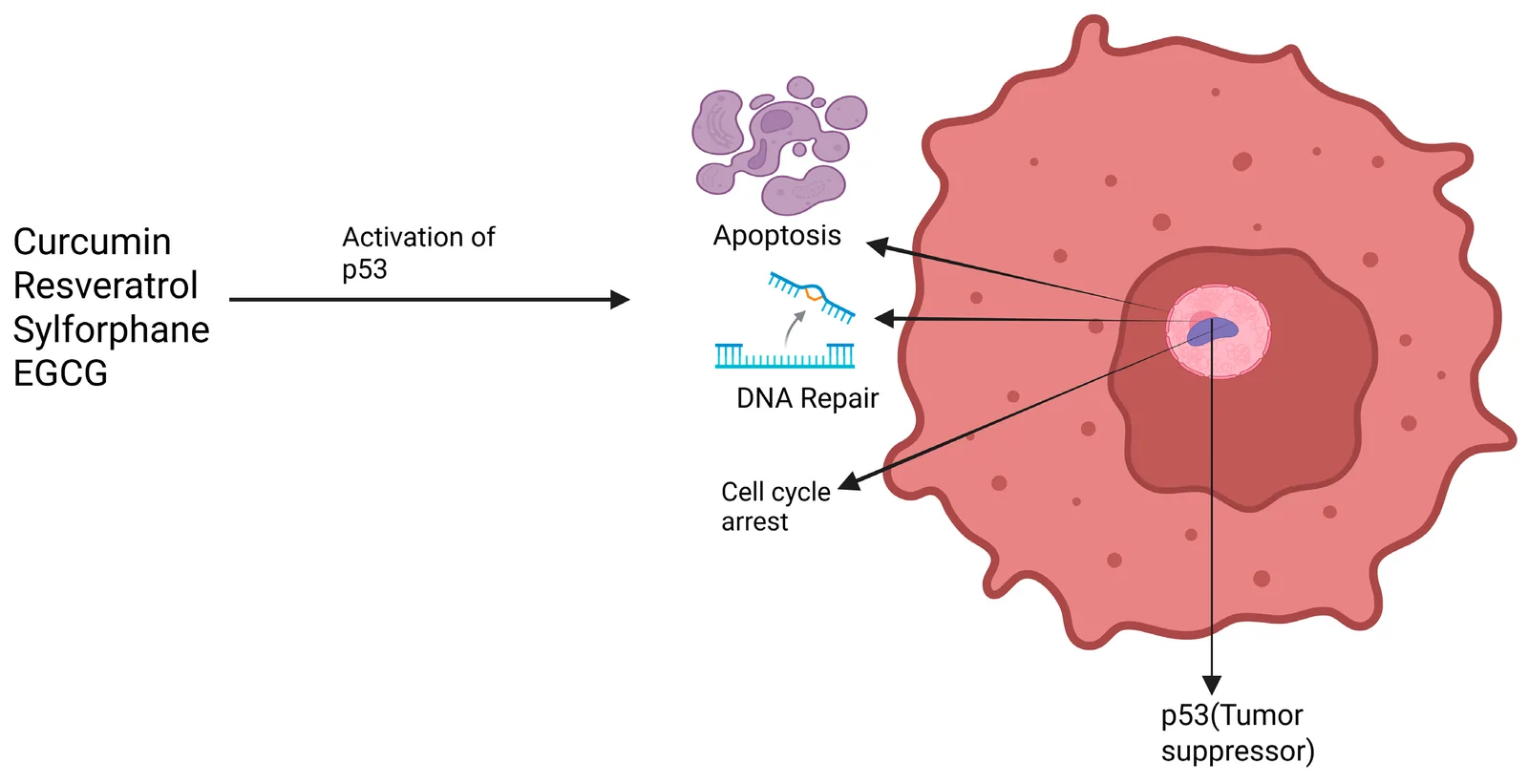
Activation of p53-dependent tumor suppressive pathways by bioactive natural compounds.

Transcriptomics has emerged as a powerful tool for understanding the impact of bioactive natural products on gene expression in cancer prevention. Unlike traditional methods that focus on individual genes, transcriptome-wide studies facilitate the identification of complex networks, pathways, and targets influenced by dietary compounds. In recent years, RNA sequencing and microarray techniques have been extensively employed to corroborate and extend findings from laboratory and animal studies, especially for compounds such as polyphenols, terpenoids, and organosulfur compounds. Polyphenols, including curcumin and RSV, have been investigated using transcriptomics to uncover their anticancer mechanisms. RNA-seq studies indicate that curcumin modulates gene activity in cancer cells, impacting genes associated with cell growth, apoptosis, stress response, and inflammation. For instance, transcriptomic analysis of curcumin-treated breast and colon cancer cells showed downregulation of genes associated with cell cycle control and cancer-related proteins, while genes involved in tumor suppression and stress response were upregulated [147]. Curcumin targets multiple pathways and can modify cancer-related gene systems. Similarly, transcriptomic studies on RSV have offered insights into its cancer-preventing properties. RNA-seq studies in hormone-sensitive and triple-negative breast cancer models demonstrate that RSV modulates gene expression in pathways related to p53, apoptosis, and inflammation, such as those regulated by NF-κB [148] (Figure 5). RSV inhibits deleterious signaling and promotes apoptosis, rendering it a promising dietary cancer preventive. Transcriptomics has also been used to study carotenoids and terpenoids, especially lycopene found in tomatoes. RNA-seq studies in prostate cancer models indicate that dietary lycopene affects genes involved in androgen signaling, antioxidant systems, and immune function. Lycopene reduces the expression of genes associated with tumor growth and progression while enhancing the expression of genes that combat oxidative stress and detoxify harmful substances [149]. These global gene expression changes support the association between carotenoid-rich diets and reduced cancer risk. In addition to polyphenols and carotenoids, transcriptomic research on organosulfur compounds, particularly sulforaphane from cruciferous vegetables, has expanded.

RNA-seq analyses indicate that sulforaphane activates protective gene networks regulated by Nrf2, while concurrently downregulating genes associated with inflammation, metastasis, and cancer stem cell characteristics. These transcriptional patterns corroborate existing knowledge regarding sulforaphane’s capacity to enhance cellular defenses and inhibit pathways that promote tumor growth, which highlights its potential in cancer prevention through dietary interventions [150]. Transcriptomic studies offer substantial scientific evidence for the mechanisms by which bioactive natural compounds exert anticancer effects. They elucidate the coordinated regulation of numerous genes and pathways, thereby linking molecular alterations to systemic effects within the body.

### 9.2. Proteomics

Proteomics facilitates the understanding of how bioactive natural compounds affect proteins within the body. Since proteins serve as the functional components of cells, their analysis allows for the evaluation of whether alterations in gene expression translate into tangible modifications in cellular function. Recent progress in mass spectrometry has enhanced the detection and quantification of proteins and their interactions in cancer models. Polyphenols, such as RSV and curcumin, have been studied through proteomics to identify the specific proteins that they influence.

Research indicates that RSV interacts with proteins that regulate cellular movement, morphology, and differentiation. In lung and breast cancer cells, proteomic analyses have shown a decrease in the activity of proteins associated with cancer metastasis and tumor growth, corroborating the hypothesis that RSV may inhibit cancer progression [151]. These findings are consistent with previous studies on the mechanisms of RSV. Similarly, curcumin has been examined using proteomics, revealing its influence on proteins involved in organelle biogenesis, mitochondrial function, and cellular stress responses.

Recent research on nano-formulated curcumin in colon cancer cells has demonstrated alterations in proteins that regulate cell growth and apoptosis, indicating enhanced efficacy when administered in this formulation [152]. These studies illustrate the capacity of proteomics to identify both direct and indirect mechanisms by which these compounds affect the body, thereby enhancing our understanding of their potential benefits in disease prevention. Furthermore, proteomics has investigated carotenoids, especially lycopene, revealing distinct patterns of protein activity associated with cancer prevention. Research on prostate and breast cancer cells indicates that lycopene influences proteins involved in maintaining intercellular chemical equilibrium, regulating cell division, and mediating hormonal responses. These findings corroborate previous studies that associated lycopene consumption with reduced tumor aggressiveness and enhanced cellular protection [153].

### 9.3. Epigenomics

Epigenomics has emerged as a significant area of focus in cancer prevention research due to its ability to elucidate how dietary intake can modulate gene function without altering the DNA sequence. Bioactive natural compounds present in the diet can influence epigenetic mechanisms, such as DNA methylation, histone modifications, and non-coding RNA regulation [154,155]. Epigenomic studies indicate that certain dietary compounds can exert long-term cancer-preventing effects by altering gene expression [154]. Among these compounds, polyphenols such as EGCG and curcumin have been extensively investigated for their epigenetic effects. EGCG is known to inhibit DNA methyltransferases, thereby reactivating tumor suppressor genes. Research in epigenetics has shown that EGCG treatment reduces methylation at gene promoters and alters DNA accessibility, underscoring its role as a natural epigenetic modifier [155]. Organosulfur compounds, especially sulforaphane, are also recognized as prominent dietary regulators of epigenetic processes.

Sulforaphane functions as a natural inhibitor of histone deacetylases, resulting in increased histone acetylation and the activation of genes involved in cancer prevention. Both laboratory and animal studies have linked sulforaphane to reduced tumor growth and decreased activity in oncogenic pathways [156]. Beyond altering DNA methylation and histone modifications, bioactive natural compounds also impact microRNA expression [154,155]. Research in epigenomics and microRNAs suggests that polyphenols and organosulfur compounds can modify microRNAs that regulate cancer-related processes, such as cell growth, proliferation, metastasis, and immune response. These findings imply that dietary compounds can influence post-transcriptional gene regulation, thereby enhancing their multifaceted impact on cancer [154,155,156].

## 10. Bioavailability and Clinical Applications

### 10.1. Bioavailability and Translational Challenges

Despite substantial mechanistic and experimental evidence supporting the anticancer potential of bioactive natural compounds, their successful translation from laboratory research to clinical application remains limited (Table 3). A primary challenge is poor bioavailability, which significantly restricts therapeutic efficacy in vivo. Many polyphenols, terpenoids, and organosulfur compounds exhibit low aqueous solubility, limited intestinal absorption, rapid first-pass metabolism, and short plasma half-lives. For instance, curcumin and RSV undergo extensive hepatic and intestinal metabolism, resulting in low systemic concentrations that are insufficient to exert sustained anticancer effects [138]. Another critical challenge is inter-individual variability, which arises due to differences in gut microbiota composition, genetic polymorphisms in metabolic enzymes, dietary habits, age, and overall health status. These factors influence the biotransformation, absorption, and bioactivity of dietary phytochemicals, leading to inconsistent responses across populations. Furthermore, many bioactive natural compounds demonstrate dose limitations, as the high concentrations required for anticancer efficacy in vitro are often not achievable through dietary intake alone and may raise concerns regarding toxicity or off-target effects when administered in concentrated forms [139]. To overcome these barriers, advanced formulation and nano-delivery strategies have gained significant attention. Nanoparticles, liposomes, solid lipid nanoparticles, micelles, and polymer-based carriers have been developed to enhance stability, solubility, and target delivery of bioactive compounds. Nano-delivery systems improve cellular uptake, prolong circulation time, and enable controlled release at tumor sites, thereby increasing therapeutic efficacy while minimizing systemic toxicity (Table 3). For example, nano-encapsulated curcumin and quercetin have demonstrated improved bioavailability and enhanced modulation of molecular targets such as PI3K/Akt, NF-κB, and apoptotic pathways in preclinical models [81]. Overall, addressing bioavailability constraints through innovative delivery systems and rational combination approaches is essential for translating bioactive natural compounds into clinically effective cancer preventive and therapeutic agents [157,158].

### 10.2. Safety, Adverse Effects, and Dose Limitations

Despite the extensive scientific evidence regarding the anticancer properties of compounds derived from natural foods, several factors impede their clinical application. First, although these compounds are generally safe for consumption as food components, research on dose optimization, pharmacokinetics, and potential adverse effects is essential before their clinical use [160]. RSV has shown significant anticancer activity against various cancer types; however, its clinical application is constrained by low bioavailability and rapid metabolism, which diminish its therapeutic efficacy. Consequently, further research is required to develop an optimal dosing regimen to enhance its effectiveness [25]. In addition, catechins, such as epigallocatechin-3-gallate, exhibit broad-spectrum anticancer activity. However, their application is restricted due to poor oral bioavailability, chemical instability, and significant inter-individual variability in pharmacokinetic properties. Addressing these issues is crucial to provide standardized formulations and design clinical studies that establish safe and effective dosing strategies [23].

Sulforaphane has shown promising outcomes in preclinical and early clinical trials. However, its application in clinical settings is hindered by limited stability, bioavailability, and the absence of standardized dosing regimens. Consequently, further research is required to optimize its dosing regimen and evaluate its long-term safety [62]. Similarly, allicin has potential therapeutic applications for diseases, but its use is constrained by its unstable chemical properties. Moreover, clinical studies lack sufficient evidence to confirm its effectiveness and safety [161].

### 10.3. Clinical Evidence

Although numerous preclinical studies have demonstrated the anticancer properties of bioactive compounds derived from foods, only a limited number have progressed to clinical evaluations. Curcumin is one of the most extensively studied compounds, having undergone Phase I, Phase II, and Phase III clinical trials, which have yielded valuable insights into its safety, pharmacokinetics and potential therapeutic applications [160]. Another compound extensively evaluated in clinical trials for its potential in cancer prevention and therapy is epigallocatechin-3-gallate (EGCG), with trials spanning from Phase I to Phase III [160] (Table 4). In addition, RSV has been subjected to clinical evaluation, focusing on safety, pharmacokinetics, and biomarker modulation. However, its clinical application remains limited due to low bioavailability [18]. Sulforaphane exhibits promising translational potential; however, further clinical trials are necessary to ascertain its efficacy [62].

**Table 4:** Provides a summary of the current clinical data for selected bioactive compounds.

Bioactive Compound	Clinical Evidence	Clinical Trial Status	Major Limitations	Reference
Sulforaphane (natural isothiocyanate)	Current evidence mainly supports chemopreventive potential through mechanistic and translational research. The reviews emphasize its promise but indicate that clinical evidence remains limited compared with curcumin, resveratrol, and EGCG	No completed late-phase cancer clinical trials are in these reviews. Clinical translation is still at an early stage	Limited stability, variable bioavailability, and insufficient standardized clinical studies remain the principal hurdle to clinical application	[62,63]
Curcumin	Clinical studies have shown curcumin in colorectal cancer, pancreatic cancer, multiple myeloma, familial adenomatous polyposis (FAP), and prevention studies	Phase I, Phase II, Phase III, pilot, recruiting, completed, suspended and terminated trials. The clinical evaluation is ongoing across multiple cancer settings rather than reporting definitive efficacy	Translation into routine clinical use remains limited because additional well-designed clinical studies are required to establish efficacy and optimize dosing	[160]
Epigallocatechin-3-gallate (EGCG)	Human studies have evaluated EGCG in lungs, bladder, breast, cervical, ovarian, oral, and other cancers for both prevention and therapy	Phase I, Phase I/II, Phase II, and Phase III trials, including recruiting, active, and completed studies	Inspire is widely studied clinically; additional large, randomized studies are required to confirm efficacy and establish optimal dosing strategies	[160]
Resveratrol	Clinical investigations include colorectal cancer and colon cancer prevention. The review describes resveratrol as a promising therapeutic and chemopreventive agent and summarizes available clinical trials	Phase I, Phase I/II, and Phase II clinical studies are reported. Most studies evaluate safety, pharmacokinetics and biomarker modulation rather than definitive clinical benefits	Poor oral bioavailability and rapid metabolism remain major barriers to successful clinical translation despite encouraging preclinical findings	[162]

## 11. Limitations

Despite substantial evidence supporting the anticancer potential of food-derived bioactive compounds through modulation of intrinsic and extrinsic apoptotic pathways, several limitations persist. Most available data originate from in vitro cell culture studies and preclinical animal models, which may not adequately represent the complexity of human tumors and their microenvironment. Clinical evidence is particularly limited for pancreatic cancer, where intrinsic apoptotic resistance and stromal barriers pose significant therapeutic challenges.

Another major limitation is the poor bioavailability, rapid metabolism, and low systemic stability of many natural compounds, such as curcumin and sulforaphane, which impede their clinical translation. Furthermore, heterogeneity in experimental designs, compound concentrations, treatment durations, and cancer models complicate direct comparison across studies. The absence of standardized biomarkers for assessing apoptosis and evaluating therapeutic responses further limits reproducibility and translational relevance. Moreover, the potential toxicity, off-target effects, and long-term safety profiles associated with high-dose or chronic exposure to these compounds remain inadequately explored in humans.

## 12. Future Directions

Future research should prioritize well-designed clinical trials to validate the efficacy, safety, and optimal dosing of bioactive compounds derived from food in cancer prevention and treatment. The development of advanced drug-delivery systems, such as nanoparticle-, liposome-, and exosome-based formulations, has the potential to significantly enhance bioavailability, tumor targeting, and therapeutic efficacy.

Investigating combination strategies that involve natural compounds with conventional chemotherapeutics, targeted agents, or immunotherapies may address chemoresistance by synergistically activating apoptotic pathways. Furthermore, identifying predictive biomarkers related to Bcl-2 family proteins, caspase activation, and death receptor signaling will facilitate patient stratification and personalized therapeutic approaches. The integration of nutrigenomics and systems biology approaches may further elucidate individual variability in response and support the integration of these compounds into precision oncology frameworks.

## 13. Conclusions

Food-derived natural products exhibit significant anticancer properties by interacting with various molecular pathways involved in the onset and progression of cancer. These compounds regulate cell cycle checkpoints, induce apoptosis, and suppress oncogenic signaling pathways. In addition, their influence on epigenetic mechanisms, oxidative stress, inflammation, angiogenesis, and metastasis enhances their role in cancer prevention. Both in vitro and in vivo studies provide evidence of their multi-targeted molecular actions with minimal toxicity. However, issues related to bioavailability and dose limitations pose challenges for clinical application. Advances in nanodelivery and formulation strategies offer promising solutions. In conclusion, natural products from foods hold significant promise for cancer prevention and the development of future therapies.

## Figures and Tables

**Table 1:** Comparison of food-derived natural compounds with conventional anticancer drugs.

Feature	Food-Derived Natural Compounds	Conventional Anticancer Drugs	Clinical Implication
Source	Dietary plants, fruits, vegetables, spices	Synthetic/semi-synthetic chemicals	Natural products have long dietary exposure
Target specificity	Multi-targeted	Single/limited targets	Reduced resistance development
Mechanism	Modulate signaling, epigenetics, redox balance	Cytotoxicity/targeted inhibition	Safer long-term modulation
Toxicity	Low to moderate	High (dose-limiting)	Better safety profile
Selectivity	Preferential to cancer cells	Affects normal rapidly dividing cells	Fewer adverse effects
Resistance	Low probability	Common (drug resistance)	Advantages in chemoprevention
Long-term use	Suitable	Limited	Ideal for prevention
Cost	Low	High	Accessible prevention strategy
Clinical application	Prevention, adjuvant therapy	Active cancer treatment	Complementary role
Evidence level	Predominantly supported by preclinical studies, with limited clinical evidence.	Supported by extensive preclinical and clinical evidence.	Additional well-designed clinical studies are required to strengthen clinical translation.
Clinical evidence	Clinical evidence is available for selected compounds	Clinical efficacy and safety have been established through extensive clinical evaluation.	Further clinical validation is needed before widespread therapeutic use
Limitations	Poor bioavailability, chemical instability, variability in therapeutic response, and lack of standardized dosing limit clinical translation.	Drug resistance, systemic toxicity, and treatment-related adverse effects may limit long-term use.	Improved formulations, standardized dosing strategies, and comprehensive clinical evaluation are required to enhance therapeutic application.

**Table 3:** Comparison of oral bioavailability, metabolism, formulation strategies, and clinical limitations of selected bioactive natural compounds.

Natural Compound	Oral Bioavailability	Major Metabolism	Formulation Strategies	Clinical Limitations	Ref
Sulforaphane	Variable oral bioavailability	Rapid metabolism	Formulation approaches to improve stability	Chemical instability and variable bioavailability	[63]
Lycopene	Low oral bioavailability	Isomerization during absorption and metabolism	Formulation approaches to improve intestinal absorption	Variable absorption and bioavailability	[63]
Allicin	Rapid degradation/poor stability	Rapid conversion to sulfur-containing metabolites	Stabilization or delivery systems to improve stability	Limited clinical evidence; lack of standardized dosing and few clinical trials	[68]
Resveratrol	Low oral bioavailability	Rapid metabolism and clearance	Formulation approaches to improve stability and bioavailability	Short plasma half-life limits clinical application	[158]
Curcumin	Low oral bioavailability	Rapid metabolism and poor systemic availability	Formulation approaches to improve bioavailability	Poor bioavailability limits clinical translation	[159]

## Data Availability

No new data were created or analyzed in this study. Data sharing is not applicable to this article.

## Abbreviations

Abbreviation	Full form
4E-BP1	4E-binding protein-1
AD	Adenocarcinoma
AMPK	AMP-activated protein kinase
AP-1	Activator protein-1
CDK	Cyclin-dependent kinase
CK1	Casein kinase 1
COX-2	Cyclooxygenase-2
DMBA	7,12-Dimethylbenz[a]anthracene
DNMT	DNA methyltransferase
DVL	Disheveled
EGCG	Epigallocatechin-3-gallate
EGFR	Epidermal growth factor receptor
EMT	Epithelial–mesenchymal transition
ER	Endoplasmic reticulum
ERK	Extracellular signal-regulated kinase
FAP	Familial adenomatous polyposis
FasL	Fas ligand
FZD	Frizzled (receptor)
GSK-3β	Glycogen synthase kinase-3 beta
HAT	Histone acetyltransferase
HDAC	Histone deacetylase
HGF	Hepatocyte growth factor
HIF-1α	Hypoxia-inducible factor-1 alpha
IGF-1	Insulin-like growth factor-1
IGF-1R	Insulin-like growth factor-1 receptor
IGFBP-3	Insulin-like growth factor-binding protein 3
IKK	IκB kinase
IL-1β	Interleukin-1 beta
IL-6	Interleukin-6
iNOS	Inducible nitric oxide synthase
IκB	Inhibitor of κB
ITCs	Isothiocyanates
JAK	Janus kinase
JNK	c-Jun N-terminal kinase
KRAS	Kirsten rat sarcoma viral oncogene
LRP5/6	Low-density lipoprotein receptor-related protein 5/6
MAPK	Mitogen-activated protein kinase
MEK	Mitogen-activated protein kinase kinase
miRNA/miR	MicroRNA
MMP	Matrix metalloproteinase
mTOR	Mammalian target of rapamycin
mTORC1/2	mTOR complex 1/2
NEMO	NF-κB essential modulator (IKKγ)
NF-κB	Nuclear factor-kappa B
PD-L1	Programmed death-ligand 1
PDK1	Phosphoinositide-dependent kinase-1
PGE2	Prostaglandin E2
PI3K	Phosphoinositide 3-kinase
PIP2	Phosphatidylinositol-4,5-bisphosphate
PIP3	Phosphatidylinositol-3,4,5-trisphosphate
PKC	Protein kinase C
PSA	Prostate-specific antigen
PTEN	Phosphatase and tensin homolog
Rb	Retinoblastoma (protein)
RNS	Reactive nitrogen species
ROS	Reactive oxygen species
RSV	Resveratrol
S6K1	S6 kinase 1
SOD	Superoxide dismutase
SQ	Squamous cell carcinoma
STAT3	Signal transducer and activator of transcription 3
TCF/LEF	T-cell factor/lymphoid enhancer factor
TGF-β	Transforming growth factor beta
TNF-α	Tumor necrosis factor alpha
TSC1/2	Tuberous sclerosis complex 1/2
VEGF	Vascular endothelial growth factor
VEGFR	Vascular endothelial growth factor receptor
Wnt	Wingless-related integration site
XIAP	X-linked inhibitor of apoptosis protein
